# Immune evasion strategy involving propionylation by the KSHV interferon regulatory factor 1 (vIRF1)

**DOI:** 10.1371/journal.ppat.1011324

**Published:** 2023-04-06

**Authors:** Jiale Shi, Xuemei Jia, Yujia He, Xinyue Ma, Xiaoyu Qi, Wan Li, Shou-Jiang Gao, Qin Yan, Chun Lu

**Affiliations:** 1 Department of Gynecology, Women’s Hospital of Nanjing Medical University, Nanjing Maternity and Child Health Care Hospital, Nanjing Medical University, Nanjing, People’s Republic of China; 2 Department of Microbiology, Nanjing Medical University, Nanjing, People’s Republic of China; 3 Key Laboratory of Pathogen Biology of Jiangsu Province, Nanjing Medical University, Nanjing, People’s Republic of China; 4 Tumor Virology Program, UPMC Hillman Cancer Center, and Department of Microbiology and Molecular Genetics, University of Pittsburgh, Pittsburgh, Pennsylvania, United States of America; University of Southern California, UNITED STATES

## Abstract

Post-translational modifications (PTMs) are essential for host antiviral immune response and viral immune evasion. Among a set of novel acylations, lysine propionylation (Kpr) has been detected in both histone and non-histone proteins. However, whether protein propionylation occurs in any viral proteins and whether such modifications regulate viral immune evasion remain elusive. Here, we show that Kaposi’s sarcoma-associated herpesvirus (KSHV)-encoded viral interferon regulatory factor 1 (vIRF1) can be propionylated in lysine residues, which is required for effective inhibition of IFN-β production and antiviral signaling. Mechanistically, vIRF1 promotes its own propionylation by blocking SIRT6’s interaction with ubiquitin-specific peptidase 10 (USP10) leading to its degradation via a ubiquitin-proteasome pathway. Furthermore, vIRF1 propionylation is required for its function to block IRF3-CBP/p300 recruitment and repress the STING DNA sensing pathway. A SIRT6-specific activator, UBCS039, rescues propionylated vIRF1-mediated repression of IFN-β signaling. These results reveal a novel mechanism of viral evasion of innate immunity through propionylation of a viral protein. The findings suggest that enzymes involved in viral propionylation could be potential targets for preventing viral infections.

## Author summary

Conventional post-translational modifications play essential roles in host antiviral immune response and viral immune evasion. Recently, a set of atypical acylations involving lysine propionylation (Kpr) have been discovered. However, whether Kpr modification occurs in viral proteins or regulates viral immune evasion remain unknown. Our study uncovers Kpr modification exists in viral interferon regulatory factor 1 (vIRF1) encoded by Kaposi’s sarcoma-associated herpesvirus (KSHV) to inhibit IFN-β production. vIRF1 promotes its own propionylation by blocking SIRT6’s interaction with ubiquitin-specific peptidase 10 (USP10) leading to its degradation via a ubiquitin-proteasome pathway. Thus, the current findings illustrate a novel mechanism of viral immune evasion strategies and provide a rationale for targeting of Kpr and the associated enzymes as an innovative therapeutic strategy of virus infections.

## Introduction

Post-translational modifications (PTMs) were discovered over 50 years ago, as a regulatory mechanism in which a protein is covalently linked to new functional groups, such as phosphate, methyl, and acetyl groups [1]. The modifications of positively charged amino acids, especially lysine, serve to regulate the activity, stability, and folding of proteins involved in a variety of cellular processes, such as metabolism, cell cycle, signal transduction and immune response [2–6]. Over the past decade, acetylation, ubiquitination and methylation are the best characterized PTMs on lysine residues. Diverse types of lysine acylation have been elucidated according to the differences in hydrocarbon chain length, hydrophobicity and charge, including propionylation (Kpr), butyrylation (Kbu), crotonylation (Kcr), hydroxyisobutyrylation (Khib), β-hydroxybutyrylation (Kbhb), malonylation (Kmal), succinylation (Ksu), and lactylation (Kla) [7]. The canonical lysine acetylation is characterized by a dynamic and reversible process modulated by specific enzymes, either “writers” or “erasers” that add or remove the acetyl group. However, it is not yet clear whether the atypical lysine PTMs in proteins are catalyzed by enzymes, and what are the “writers” and “erasers”? Moreover, the functional roles of these novel lysine PTMs have been minimally characterized.

To initiate infection, viruses consume host cellular resources to replicate, which may also involve cellular lysine PTMs. The PTMs of host proteins activate innate immunity to control viral replication; meanwhile, the PTMs of viral proteins endow them the ability to evade innate immunity through regulating the compartmentalization, trafficking, and physical interaction of key molecules involved in immunological processes. For instance, viral interferon regulatory factor 1 (vIRF1) encoded by Kaposi’s sarcoma-associated herpesvirus (KSHV) interacts with ubiquitin-specific protease 7 (USP7) to repress the p53 enzymatic activities, thus inhibiting p53-mediated antiviral responses [8]. However, whether viruses, such as KSHV, can evade host immune response through novel lysine PTMs remains unknown.

KSHV, also known as human herpesvirus 8 (HHV-8), is a large double stranded DNA (dsDNA) virus initially detected in a Kaposi’s sarcoma (KS) lesion from an AIDS patient in 1994 [9]. In addition to KS, KSHV is also associated with primary effusion lymphoma (PEL), a subset of multicentric Castleman’s disease (MCD), and KSHV-associated inflammatory cytokine syndrome (KICS) [10,11,12]. Like other herpesviruses, KSHV establishes a persistent infection within the host through two distinct phases, including a latent phase and a productive lytic replication phase. During the latent phase, the viral genome is maintained as episome expressing only a minimal number of viral genes, which enables the virus to persist and evade host immunity. During the lytic phase, most viral genes are expressed and the virus utilizes multiple immunomodulatory strategies to sustain replication, including expression of viral homologs of cellular interferon (IFN) regulatory factors (vIRFs).

Containing an N-terminal DNA-binding domain (DBD) and a C-terminal IRF interaction domain (IAD), vIRF1 is the most studied of the four KSHV vIRFs [13]. It shares 26.6% and 26.2% of protein homologs with human IRF3 and IRF7, respectively [13]. However, unlike its cellular homologs, vIRF1 cannot bind to DNA directly, but still exhibits inhibitory effects on IFN-mediated signaling by interacting with cellular proteins [14,15]. It is generally agreed that vIRF1 interacts with IRF3 to disrupt the formation of IRF3-CBP/p300 complexes, resulting in the suppression of the early response of IFN to virus infection [16]. The DNA sensing via the cGMP-AMP synthase (cGAS) and stimulator of IFN-dependent genes (STING) pathway is a powerful innate immune response to DNA viruses. vIRF1 represses the cGAS-STING pathway at multiple nodes to inhibit IFN-β production [17]. However, whether the novel lysine PTMs are involved in vIRF1-related host IFN response has not been verified.

In this study, we showed that vIRF1 underwent lysine propionylation (Kpr) at Lys406 and Lys442 to downregulate IFN-β production in response to virus infection. Among the sirtuins family responsible for removing acyl-lysine modifications, SIRT6 played a counteractive role in vIRF1 propionylation, and its association with ubiquitin-specific peptidase 10 (USP10) could be inhibited by vIRF1 by targeting it for degradation. Mechanically, SIRT6-mediated vIRF1 propionylation inhibited IFN-related innate immunity by blocking IRF3-CBP/p300 recruitment, as well as the STING DNA sensing pathway. Here we propose a novel mechanism by which a viral homolog of cellular IRF hijacks the cellular lysine propionylation system to facilitate immune evasion. This study provides insights into the immune evasion strategies of KSHV.

## Results

### KSHV vIRF1 is propionylated at Lys406 and Lys442 to repress antiviral response

**Fig 1:**
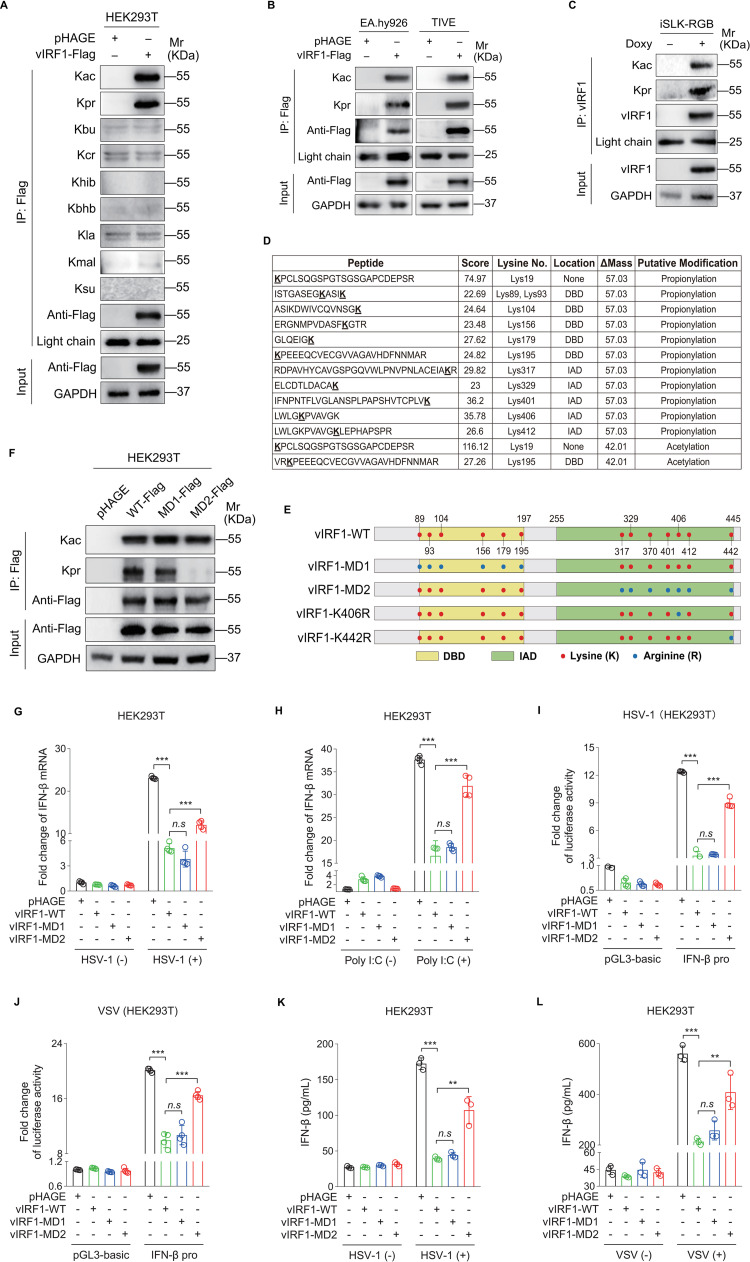
vIRF1 is propionylated by lysine to repress IFN-β production. **(A).** HEK293T cells were transduced with lentiviral vIRF1 (**vIRF1-Flag**) or control lentivirus (**pHAGE**), and then subjected to anti-Flag immunoprecipitation assay (IP). Landscape of lysine acylation in the immunoprecipitated vIRF1 was examine with pan-antibodies specific to lysine acetylation (**Kac**), propionylation (**Kpr**), butyrylation (**Kbu**), crotonylation (**Kcr**), hydroxyisobutyrylation (**Khib**), β-hydroxybutyrylation (**Kbhb**), lactylation (**Kla**), malonylation (**Kmal**), and succinylation (**Ksu**), respectively. **(B).** EA.hy926 and TIVE cells transduced by lentiviral vIRF1 (**vIRF1-Flag**) or its control (**pHAGE**) were subjected to the anti-Flag immunoprecipitation. The immuno-isolated proteins were analyzed by Western blot using anti-acetyllysine (**Kac**) and anti-propionyllysine (**Kpr**) antibodies, respectively. **(C).** iSLK-RGB cells induced with or without Doxycycline (**Doxy**) for 48 h were subjected to the anti-vIRF1 immunoprecipitation, and the precipitated proteins were examined by anti-acetyllysine (**Kac**) and anti-propionyllysine (**Kpr**) antibodies, respectively. **(D).** HEK293T cells transduced with lentiviral vIRF1 were subjected to anti-Flag immunoprecipitation, SDS-PAGE, in-gel digestion, and LC-MS/MS analysis. Sequence of identified peptides, score of peptide segment for matching degree, and the number and location of the identified lysines (**K**) in mass spectrometry analysis were shown. The increased 57.03 and 42.01 Da on lysine (**K**) could be explained as propionylation and acetylation, respectively. **(E).** The lysine (**K**) sites located in the DNA-binding domain (**DBD**) and the IRF interaction domain (**IAD**) of wild type vIRF1 (**vIRF1-WT**) were marked in red, of which were mutated to arginine (**R**) were marked in blue. The DBD-mutated, IAD-mutated, and Lys406 (K406)- or Lys442 (K442)-mutated vIRF1 were named as **vIRF1-MD1**, **vIRF1-MD2**, **vIRF1-K406R** and **vIRF1-K442R**, respectively. **(F).** HEK293T cells transduced with the wild type vIRF1 (**WT-Flag**), DBD-mutated vIRF1 (**MD1-Flag**), IAD-mutated vIRF1 (**MD2-Flag**), or its control (**pHAGE**) were subjected to the anti-Flag immunoprecipitation. The precipitated proteins were analyzed by Western blot using anti-acetyllysine (**Kac**) and anti-propionyllysine (**Kpr**) antibodies, respectively. **(G-H).** HEK293T cells transduced with the wild type vIRF1 (**vIRF1-WT**), DBD-mutated vIRF1 (**vIRF1-MD1**), IAD-mutated vIRF1 (**vIRF1-MD2**), or its control (**pHAGE**) were infected with HSV-1 for 16 h (**G**) or transfected with Poly I:C for 8 h (**H**). Cells were harvested for the measurement of IFN-β mRNA levels by RT-qPCR. ***, *P* < 0.001 by Student’s *t* test; *n*.*s*, not significant. **(I-J).** HEK293T cells transduced with the wild type vIRF1 (**vIRF1-WT**), DBD-mutated vIRF1 (**vIRF1-MD1**), IAD-mutated vIRF1 (**vIRF1-MD2**), or its control (**pHAGE**) were transfected with IFN-β promoter luciferase reporter plasmid (**IFN-β pro**) or the control (**pGL3-basic**) for 24 h. Cells were further infected with HSV-1 (**I**) or VSV (**J**) for 16 h and examined for IFN-β promoter luciferase activity. ***, *P* < 0.001 by Student’s *t* test; *n*.*s*, not significant. **(K-L).** HEK293T cells transduced with the wild type vIRF1 (**vIRF1-WT**), DBD-mutated vIRF1 (**vIRF1-MD1**), IAD-mutated vIRF1 (**vIRF1-MD2**), or its control (**pHAGE**) were infected with HSV-1 (**K**) or VSV (**L**) for 16 h. The IFN-β protein levels from cells supernatants were examined by ELISA. **, *P* < 0.01 and ***, *P* < 0.001 by Student’s *t* test; *n*.*s*, not significant.

To investigate the post-translational modifications (PTMs) of viral protein, immunoprecipitation (IP) assay was performed to analyze lysine acetylation (Kac), propionylation (Kpr), butyrylation (Kbu), crotonylation (Kcr), hydroxyisobutyrylation (Khib), β-hydroxybutyrylation (Kbhb), lactylation (Kla), malonylation (Kmal) and succinylation (Ksu) on KSHV vIRF1 with a various of pan-acyl-lysine antibodies. We found that vIRF1 was modified by both acetylation and propionylation but not other acyl-lysine modifications in HEK293T cells (Fig 1A). Since KS tumor cells express endothelial cell markers, the same experiments were performed in endothelial cell lines EA.hy926 and TIVE. We observed both acetylation and propionylation of vIRF1 in these cells (Fig 1B), implying that the PTMs pattern of vIRF1 is independent of cell type. To examine whether the above findings could be confirmed in the context of viral infection, we induced a KSHV infected cell line iSLK-RGB with doxycycline to trigger the expression of KSHV lytic genes, including vIRF1. Immunoprecipitation assay (IP) demonstrated the presence of both propionylation and acetylation modifications on endogenous vIRF1 after KSHV reactivation (Fig 1C).

To determine which lysine residue mediates vIRF1 acetylation and propionylation, we transduced vIRF1-expressing plasmid into HEK293T cells, and then isolated vIRF1 by immunoprecipitation for LC-MS/MS analysis to map sites of lysine acetylation and propionylation. Consistent with the results of IP analysis (Fig 1A–1C), we identified 12 lysine propionylation sites and 2 acetylation sites in vIRF1 (Fig 1D). Noticeably, most predicted propionyl-lysines of vIRF1 were located in the functional domains.

To confirm the results from mass spectrometry, all lysine residues within the N-terminal DNA-binding domain (DBD) and the C-terminal IRF interaction domain (IAD) of vIRF1 were mutated to arginine, named as MD1 and MD2, respectively, to mimic its positive charge state *in vivo* (Fig 1E). Mutations in the IAD (MD2), rather than the DBD (MD1), completely abolished vIRF1 propionylation; however, mutations at lysine in both domains did not affect the acetylation level of vIRF1 (Fig 1F). Previous studies have shown that the primary and most important function of vIRF1 is blocking IFN-β production in the antiviral response [16,17]. To illustrate whether KSHV evasion of immune response depends on vIRF1 propionylation, HEK293T cells expressing wild type (WT), DBD mutant (MD1) or IAD mutant (MD2) of vIRF1 were infected with herpes simplex virus type 1 (HSV-1) or treated with a synthetic analog of viral double stranded RNA (Poly I:C). RT-qPCR showed that ectopic expression of vIRF1 inhibited the mRNA level of IFN-β. However, lysine mutations in the IAD (MD2), but not the DBD (MD1) abolished the vIRF1 inhibitory effect (Fig 1G and 1H). Similarly, vIRF1 overexpression suppressed the IFN-β promoter activity during HSV-1 or vesicular stomatitis virus (VSV) infection in a luciferase reporter assay, and this inhibitory effect was abolished following mutations of lysine to arginine in the IAD (MD2), but not the DBD (MD1) (Fig 1I and 1J). Consistently, mutations of lysine in IAD resulted in a higher level of IFN-β protein upon the infection by HSV-1 or VSV (Fig 1K and 1L).

**Fig 2:**
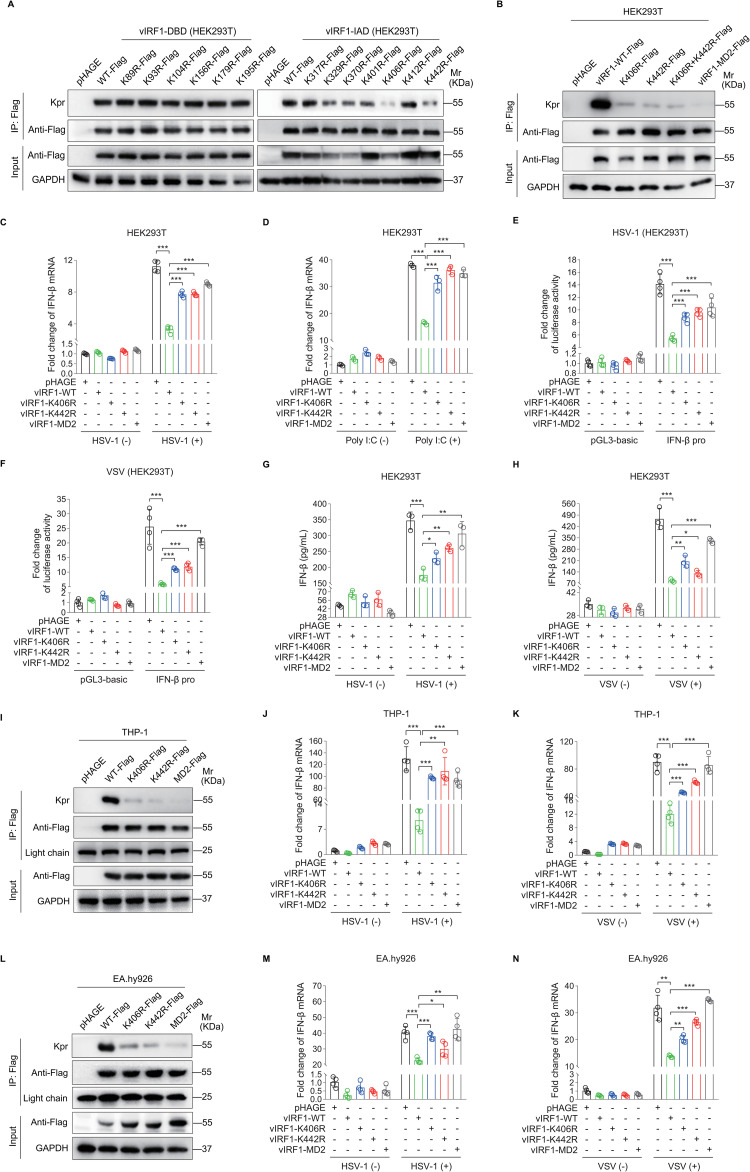
Propionyllysines in vIRF1 are identified in IFN antiviral response. **(A).** HEK293T cells transduced with lentiviral wild type vIRF1 (**WT-Flag**), the mutant forms (**K89R-Flag**, **K93R-Flag**, **K104R-Flag**, **K156R-Flag**, **K179R-Flag**, **K195R-Flag**) in DBD (**vIRF1-DBD**), the mutant forms (**K317R-Flag**, **K329R-Flag**, **K370R-Flag**, **K401R-Flag**, **K406R-Flag**, **K412R-Flag**, **K442R-Flag**) in IAD (**vIRF1-IAD**), or its control (**pHAGE**) were subjected to the anti-Flag immunoprecipitation, and the precipitated proteins were examined by Western blot with anti-propionyllysine (**Kpr**) antibodies. **(B).** HEK293T cells transduced with lentiviral wild type vIRF1 (**vIRF1-WT-Flag**), the mutant forms (**K406R-Flag**, **K442R-Flag**, **K406R+K442R-Flag**) in IAD, IAD-mutated vIRF1 (**vIRF1-MD2-Flag**), or its control (**pHAGE**) were subjected to the anti-Flag immunoprecipitation. The precipitated proteins were analyzed by Western blot using anti-propionyllysine (**Kpr**) antibodies. **(C-D).** HEK293T cells transduced with lentiviral wild type vIRF1 (**vIRF1-WT**), mutant vIRF1 (**vIRF1-K406R**, **vIRF1-K442R**, **vIRF1-MD2**), or its control (**pHAGE**) were infected with HSV-1 for 16 h (**C**) or transfected with Poly I:C for 8 h (**D**) before IFN-β mRNA levels were measured by RT-qPCR. ***, *P* < 0.001 by Student’s *t* test. **(E-F).** HEK293T cells with the wild type vIRF1 (**vIRF1-WT**), mutant vIRF1 (**vIRF1-K406R**, **vIRF1-K442R**, **vIRF1-MD2**), or its control (**pHAGE**) overexpression were co-transfected with IFN-β promoter luciferase reporter plasmid (**IFN-β pro**) or the control (**pGL3-basic**) for 24 h. The indicated cells were further infected with HSV-1 (**E**) or VSV (**F**) for 16 h before IFN-β promoter luciferase activity were examined. ***, *P* < 0.001 by Student’s *t* test. **(G-H).** HEK293T cells transduced with lentiviral wild type vIRF1 (**vIRF1-WT**), mutant vIRF1 (**vIRF1-K406R**, **vIRF1-K442R**, **vIRF1-MD2**), or its control (**pHAGE**) were infected with HSV-1 (**G**) or VSV (**H**) for 16 h. The IFN-β protein levels from cells supernatants were examined by ELISA. *, *P* < 0.05; **, *P* < 0.01; ***, *P* < 0.001 by Student’s *t* test. **(I).** THP-1 cells transduced with lentiviral wild type vIRF1 (**WT-Flag**), the mutant forms (**K406R-Flag**, **K442R-Flag**, **MD2-Flag**), or its control (**pHAGE**) were subjected to the anti-Flag immunoprecipitation. The precipitated proteins were analyzed by Western blot using anti-propionyllysine (**Kpr**) antibodies. **(J-K).** THP-1 cells transduced with the wild type vIRF1 (**WT-Flag**), the mutant forms (**K406R-Flag**, **K442R-Flag**, **MD2-Flag**), or its control (**pHAGE**) were further infected with HSV-1 (**J**) or VSV (**K**) for 16 h before IFN-β mRNA levels were measured by RT-qPCR. **, *P* < 0.01; ***, *P* < 0.001 by Student’s *t* test. **(L).** EA.hy926 cells transduced with the wild type vIRF1 (**WT-Flag**), the mutant forms (**K406R-Flag**, **K442R-Flag**, **MD2-Flag**), or its control (**pHAGE**) were subjected to the anti-Flag immunoprecipitation. The precipitated proteins were analyzed by Western blot using anti-propionyllysine (**Kpr**) antibodies. **(M-N).** EA.hy926 cells transduced with the wild type vIRF1 (**WT-Flag**), the mutant forms (**K406R-Flag**, **K442R-Flag**, **MD2-Flag**), or its control (**pHAGE**) were infected with HSV-1 (**M**) or VSV (**N**) for 16 h before IFN-β mRNA levels were examined by RT-qPCR. *, *P* < 0.05; **, *P* < 0.01; ***, *P* < 0.001 by Student’s *t* test.

To further determine the lysine residues responsible for vIRF1 propionylation, the single lysine residue was mutated to arginine in vIRF1 IAD domain (Fig 1E). Consistent with the results of mass spectrometry (Fig 1D and S1 Fig), mutation of Lys406 in the IAD but not other predicted lysines in vIRF1, to arginine (K406R) decreased the level of vIRF1 propionylation in HEK293T cells (Fig 2A). Surprisingly, mutation of Lys442 to arginine (K442R) also reduced vIRF1 propionylation (Fig 2A) albeit Lys442 propionylation was not observed in the mass spectrum (Figs 1D and S1). However, dual-mutation of Lys406 and Lys442 did not exhibit any synergistic effects on vIRF1 propionylation (Fig 2B). To explore the role of Lys406 and Lys442-mediated vIRF1 propionylation in IFN-β production, we examined the mRNA and protein levels of IFN-β, and its promoter activity by RT-qPCR, ELISA, and luciferase reporter assay, respectively. Similar to mutations of all lysine residues in the IAD, mutation of K406R or K442R alone abolished the inhibitory effect of vIRF1 on the mRNA and protein levels of IFN-β, and its promoter activity compared with WT vIRF1 (Fig 2C–2H). Similar results were observed in a leukemia monocytic cell line THP-1 (Fig 2I–2K) and an endothelial cell line EA.hy926 (Fig 2L–2N), while dual-mutations of Lys406 and Lys442 did not further inhibit vIRF1’s function in blocking IFN-β production (S2 Fig).

Taken together, these results suggest that vIRF1 lysine propionylation at Lys406 and Lys442 decreases IFN-β production.

### vIRF1 induces SIRT6 degradation to promote self-propionylation and immune evasion

**Fig 3:**
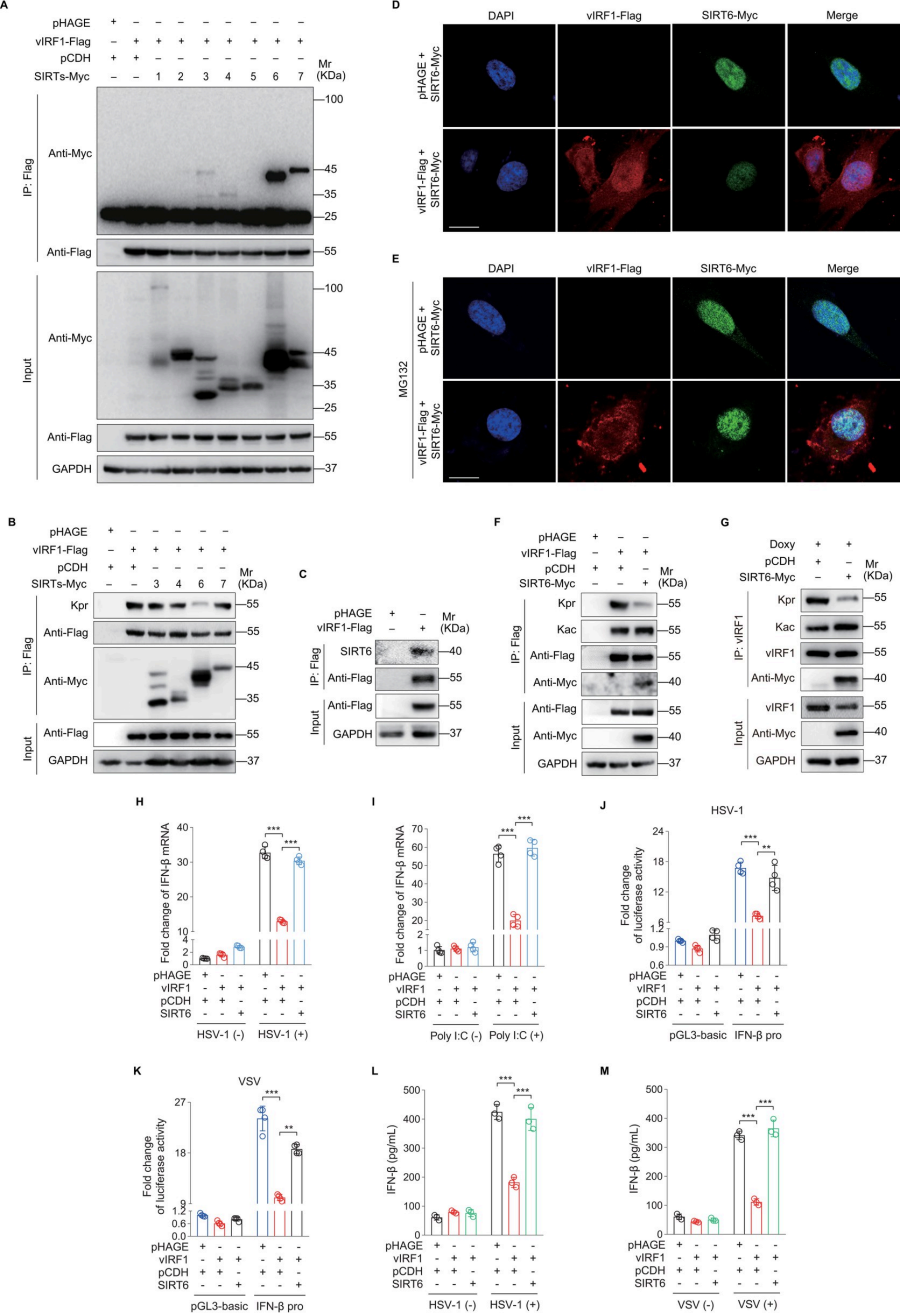
SIRT6 depropionylates vIRF1 to induce IFN-β production. **(A).** HEK293T cells transduced with lentiviral vIRF1 (**vIRF1-Flag**) or its control (**pHAGE**) were infected with lentiviral sirtuins (**SIRTs-Myc 1~7**) or its control (**pCDH**), and then subjected to anti-Flag immunoprecipitation. The interaction between vIRF1 and SIRT1~SIRT7 proteins was examined with anti-Myc antibody. **(B).** HEK293T cells transduced with lentiviral vIRF1 (**vIRF1-Flag**) or its control (**pHAGE**) were infected with lentiviral sirtuins (**SIRTs-Myc 3/4/6/7**) or its control (**pCDH**), and then subjected to anti-Flag immunoprecipitation. The immuno-isolated proteins were analyzed by Western blot using anti-propionyllysine (**Kpr**) antibody. **(C).** HEK293T cells were transduced with lentiviral vIRF1 (**vIRF1-Flag**) or its control (**pHAGE**), and then subjected to anti-Flag immunoprecipitation. The precipitated proteins were analyzed by Western blot using anti-SIRT6 antibody. **(D).** EA.hy926 cells transduced with lentiviral vIRF1-Flag and SIRT6-Myc (**vIRF1-Flag + SIRT6-Myc**) were employed to detect the expression of vIRF1 and SIRT6 by immunofluorescence staining. **(E).** EA.hy926 cells transduced with lentiviral vIRF1-Flag and SIRT6-Myc (**vIRF1-Flag + SIRT6-Myc**) were further treated with MG132 (5 μM) for 24 h, and then were employed to examine the colocalization of vIRF1 and SIRT6 by immunofluorescence staining. **(F).** HEK293T cells transduced with lentiviral vIRF1 (**vIRF1-Flag**) or its control (**pHAGE**) were infected with lentiviral SIRT6 (**SIRT6-Myc**) or its control (**pCDH**), and then subjected to anti-Flag immunoprecipitation. The immuno-isolated proteins were analyzed by Western blot using anti-propionyllysine (**Kpr**) and anti-acetyllysine (**Kac**) antibodies, respectively. **(G).** iSLK-RGB cells transduced with lentiviral SIRT6 (**SIRT6-Myc**) or its control (**pCDH**) and induced with Doxycycline (**Doxy**) for 48 h were subjected to the anti-vIRF1 immunoprecipitation, and the precipitated proteins were examined by anti-propionyllysine (**Kpr**) and anti-acetyllysine (**Kac**) antibodies, respectively. **(H-I).** vIRF1-expressing HEK293T cells were transduced with lentiviral SIRT6 (**SIRT6**) or its control (**pCDH**), and further infected with HSV-1 for 16 h (**H**) or transfected with Poly I:C for 8 h (**I**) before IFN-β mRNA levels were measured by RT-qPCR. ***, *P* < 0.001 by Student’s *t* test. **(J-K).** vIRF1-expressing HEK293T cells were transduced with lentiviral SIRT6 (**SIRT6**) or its control (**pCDH**), and further co-transfected with IFN-β promoter luciferase reporter plasmid (**IFN-β pro**) or the control (**pGL3-basic**) for 24 h. The indicated cells were further infected with HSV-1 (**J**) or VSV (**K**) for 16 h before IFN-β promoter luciferase activity were measured. **, *P* < 0.01; ***, *P* < 0.001 by Student’s *t* test. **(L-M).** vIRF1-expressing HEK293T cells were transduced with lentiviral SIRT6 (**SIRT6**) or its control (**pCDH**), and further infected with HSV-1 (**L**) or VSV (**M**) for 16 h. The IFN-β protein levels from cells supernatants were measured by ELISA. ***, *P* < 0.001 by Student’s *t* test.

It is reported that lysine acetylation is reversibly and dynamically regulated by histone acetyltransferases (HATs) and histone deacetylases (HDACs), which add and remove acetyl-Coenzyme A (acetyl-CoA) as “writers” and “erasers”, respectively [18,19]. Emerging evidence suggested that the NAD^+^-dependent sirtuins (SIRT1~7) could remove acyl groups besides acetyl from lysine residues [20]. To determine the critical sirtuin members that primarily depropionylate the lysine residues in vIRF1, co-immunoprecipitation (Co-IP) assay was performed in HEK293T cells. We found that vIRF1 interacted with several specific sirtuins, including SIRT3, SIRT4, SIRT6 and SIRT7 (Fig 3A). However, only SIRT6 overexpression decreased the level of propionylated vIRF1 (Fig 3B). vIRF1 interacted with endogenous SIRT6 (Fig 3C). Immunofluorescence staining assay (IFA) showed that vIRF1-Flag not only reduced the SIRT6-Myc expression level in endothelial cells (Fig 3D) but also exhibited colocalization with SIRT6-Myc (Fig 3E). To confirm that SIRT6 reduced vIRF1 lysine propionylation, we co-expressed vIRF1 with SIRT6 in HEK293T cells, and examined vIRF1 lysine propionylation. Overexpression of SIRT6 reduced the propionylation but not acetylation of exogenous and endogenous vIRF1 (Fig 3F and 3G). SIRT6 overexpression also significantly blocked vIRF1-repression of IFN-β production upon HSV-1 infection or Poly I:C treatment (Fig 3H and 3I), as well as activation of IFN-β promoter activity measured by luciferase reporter assay (Fig 3J and 3K). SIRT6 relieved vIRF1 inhibition of IFN-β protein expression in HEK293T cells (Fig 3L and 3M). These results indicate that SIRT6 mediates vIRF1 depropionylation to induce IFN-β production.

**Fig 4:**
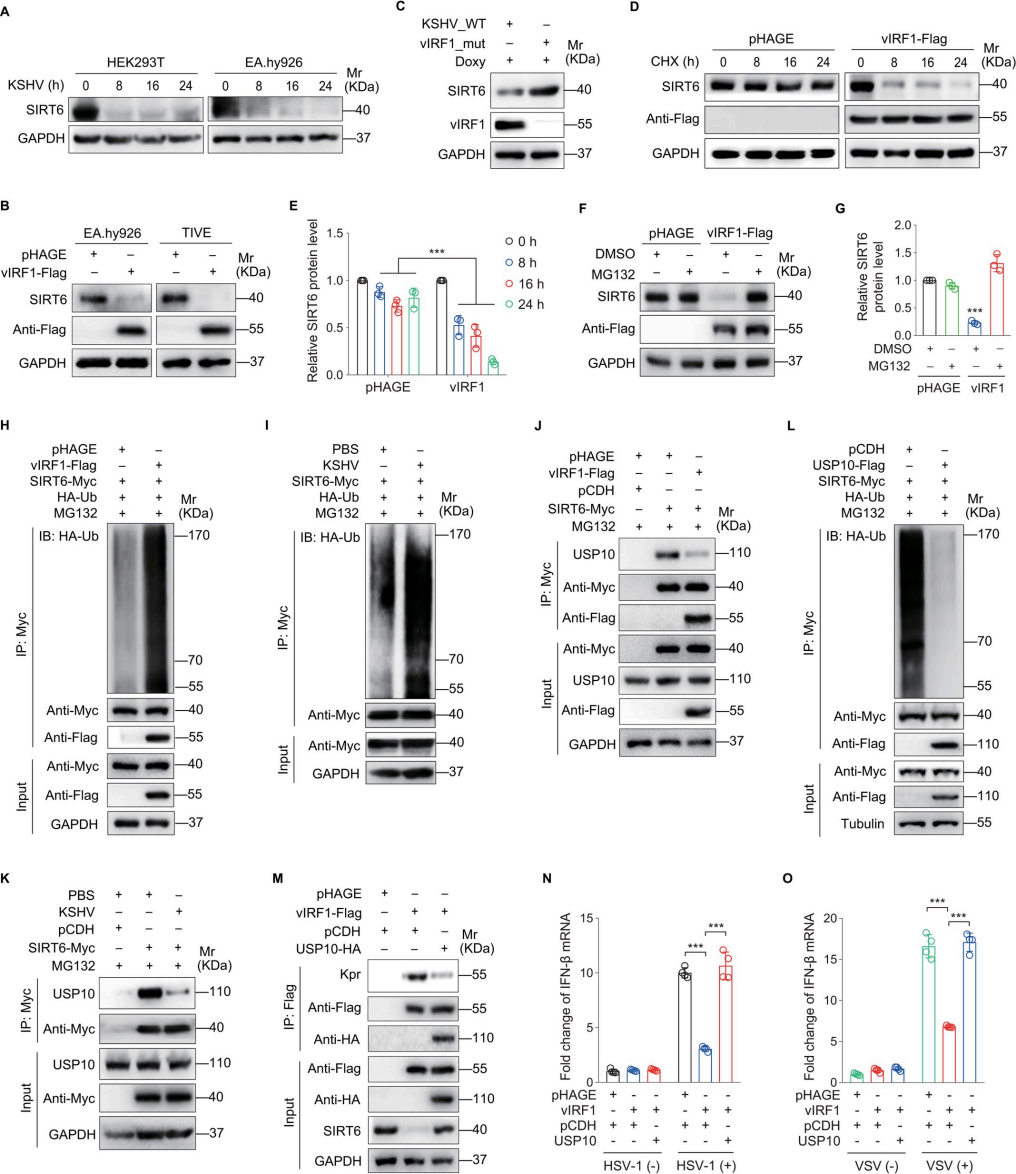
vIRF1 promotes SIRT6 degradation by disrupting the recruitment of its deubiquitinase USP10. **(A).** The protein expression levels of SIRT6 in HEK293T and EA.hy926 cells infected with KSHV for 0, 8, 16, 24 h were examined by Western blot. **(B).** The protein expression levels of SIRT6 in EA.hy926 and TIVE cells transduced by lentiviral vIRF1 (**vIRF1-Flag**) or its control (**pHAGE**) were analyzed by Western blot. **(C).** The protein expression levels of SIRT6 in iSLK-RGB (**KSHV_WT**) and K9_mutant iSLK-RGB (**vIRF1_mut**) cells were analyzed by Western blot after doxycycline (**Doxy**) treatment for 48 h. **(D).** HEK293T cells transduced by lentiviral vIRF1 (**vIRF1-Flag**) or its control (**pHAGE**) were treated with CHX (10 μg/mL) for 0, 8, 16, 24 h. The protein expression levels of SIRT6 in the indicated cells were detected by Western blot to monitor its stability. **(E).** Results were quantified in (**D**). ***, *P* < 0.001 by Student’s *t* test. **(F).** HEK293T cells transduced by lentiviral vIRF1 (**vIRF1-Flag**) or its control (**pHAGE**) were treated with MG132 (**MG132;** 5 μM) or its control (**DMSO**) for 24 h. The expression levels of SIRT6 in the indicated cells were examined by Western blot to verify its degradation pathway. **(G).** Results were quantified in (**F**). ***, *P* < 0.001 by Student’s *t* test. **(H).** HEK293T cells treated as in (**F**) were transfected with the HA-Ub and SIRT6-Myc constructs, and then subjected to anti-Myc immunoprecipitation for detection of SIRT6 ubiquitination. **(I).** HEK293T cells with HA-Ub and SIRT6-Myc transfection were treated with MG132 (5 μM) for 24 h, and then infected with KSHV (**KSHV**) or its control (**PBS**) for another 24 h. The indicated cells were subjected to anti-Myc immunoprecipitation for detection of SIRT6 ubiquitination. **(J).** HEK293T cells transduced by lentiviral vIRF1 (**vIRF1-Flag**) or its control (**pHAGE**) were transfected with the SIRT6 plasmid (**SIRT6-Myc**) or its control (**pCDH**), and treated with MG132 (5 μM) for 24 h. The indicated cells were then subjected to anti-Myc immunoprecipitation and analyzed by Western blot using anti-USP10 antibody. **(K).** HEK293T cells infected with KSHV (**KSHV**) or its control (**PBS**) were transfected with the SIRT6 plasmid (**SIRT6-Myc**) or its control (**pCDH**), and treated with MG132 (5 μM) for 24 h. The indicated cells were then subjected to anti-Myc immunoprecipitation and analyzed by Western blot using anti-USP10 antibody. **(L).** HEK293T cells with co-transfection of HA-Ub and SIRT6-Myc plasmids were transfected with USP10 plasmid (**USP10-Flag**) or its control (**pCDH**), and then treated with MG132 (5 μM) for 24 h. The indicated cells were subjected to anti-Myc immunoprecipitation for detection of SIRT6 ubiquitination. **(M).** HEK293T cells transduced by lentiviral vIRF1 (**vIRF1-Flag**) or its control (**pHAGE**) were transfected with the USP10 plasmid (**USP10-HA**) or its control (**pCDH**). Cells were subjected to anti-Flag immunoprecipitation and analyzed by Western blot using anti-propionyllysine (**Kpr**) antibodies. **(N-O).** HEK293T cells treated as in (**M**) were infected with HSV-1 (**N**) or VSV (**O**) for 16 h before IFN-β mRNA levels were measured by RT-qPCR. ***, *P* < 0.001 by Student’s *t* test.

To gain further insights into the mechanism of SIRT6-mediated vIRF1 propionylation in immune evasion during KSHV infection, we examined the expression level of SIRT6. Both KSHV infection and vIRF1 overexpression decreased SIRT6 protein level (Figs 4A, 4B and S3), but not its mRNA level (S4A and S4B Fig), while deletion of vIRF1 in KSHV-iSLK-RGB cells increased SIRT6 protein level (Fig 4C). Treatment with cycloheximide (CHX), a *de novo* protein biosynthesis inhibitor, increased SIRT6 degradation rate in vIRF1-expressing cells (Fig 4D and 4E), indicating that vIRF1 downregulated SIRT6 by promoting its degradation. In contrast, treatment with MG132, a ubiquitin-proteasome pathway inhibitor, effectively blocked vIRF1-induced SIRT6 degradation (Fig 4F and 4G). Consistent with these results, overexpression of vIRF1 or KSHV infection increased SIRT6 polyubiquitylation (Fig 4H and 4I), indicating that vIRF1 accelerated SIRT6 degradation via the proteasome-dependent pathway.

Previous studies have shown that ubiquitin-specific peptidase 10 (USP10) interacts with SIRT6 and suppresses its ubiquitination and degradation [21,22]. We therefore investigated whether vIRF1 mediated SIRT6 degradation was involved with USP10. USP10 overexpression increased SIRT6 protein expression (S5 Fig) while USP10 knockdown had the opposite effect (S6 Fig). Neither vIRF1 overexpression nor KSHV infection had any effect on USP10 expression (S7 Fig). However, both vIRF1 overexpression and KSHV infection interfered the interaction of USP10 with SIRT6 (Fig 4J and 4K). To confirm that USP10 mediates SIRT6 deubiquitination, SIRT6-Myc was immunoprecipitated in USP10-expressing cells. The results revealed that USP10 negatively regulated SIRT6 ubiquitination (Fig 4L). We further determined the role of USP10 in vIRF1 propionylation and inhibition of IFN-β production. Overexpression of USP10 not only repressed vIRF1 propionylation (Fig 4M) but also rescued vIRF1 inhibition of IFN-β mRNA level (Fig 4N and 4O). Collectively, these results suggest that by blocking the interaction between USP10 and SIRT6, vIRF1 promotes SIRT6 degradation via an ubiquitin-proteasome pathway resulting in self-propionylation and inhibition of IFN-β production.

### Propionylated vIRF1 represses IFN antiviral response by blocking IRF3-CBP/p300 recruitment and the STING DNA sensing pathway

**Fig 5:**
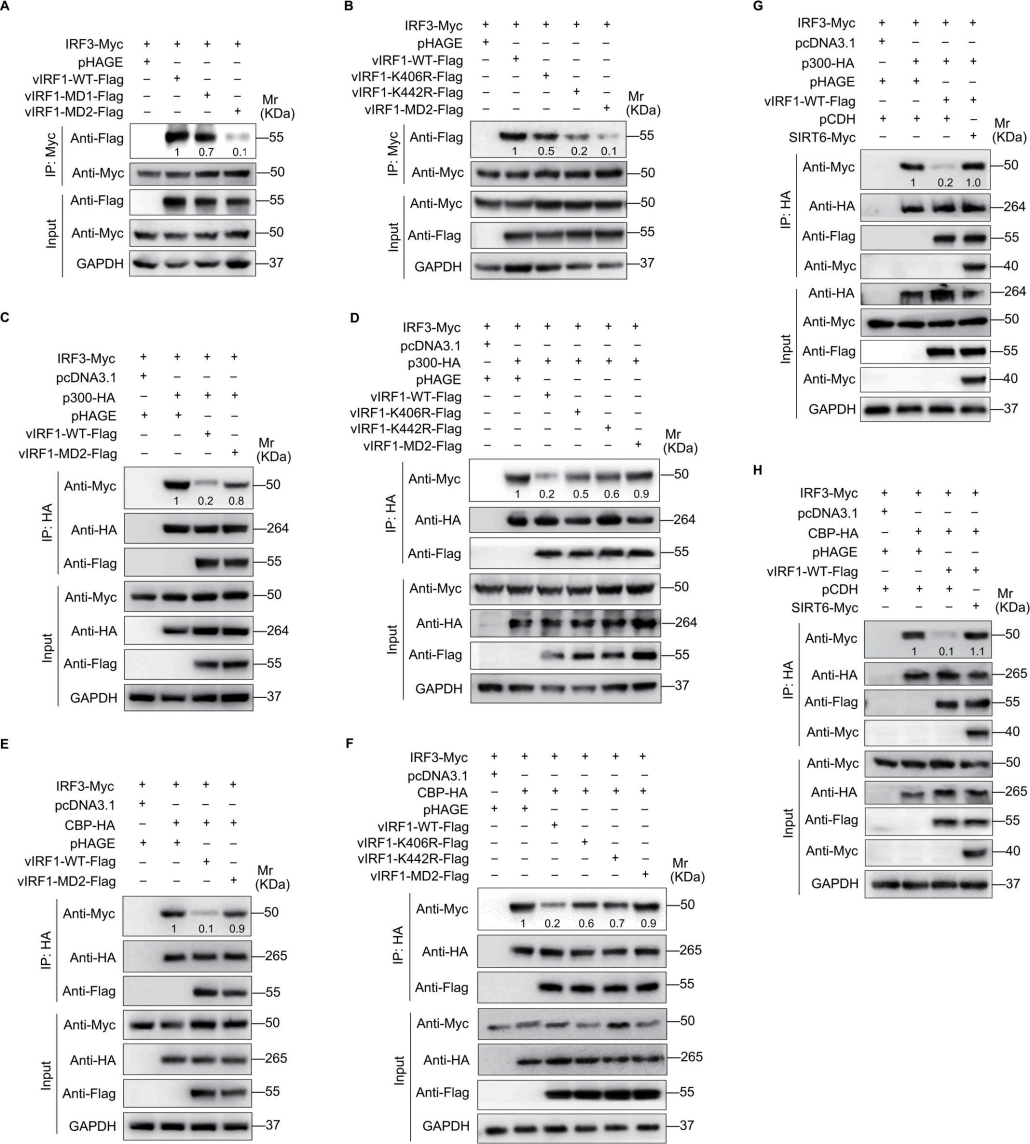
vIRF1 propionylation blocks the formation of IRF-3-CBP/p300 complexes. **(A).** HEK293T cells with IRF3 overexpression (**IRF3-Myc**) were transfected with the wild type vIRF1 (**vIRF1-WT-Flag**), DBD-mutated vIRF1 (**vIRF1-MD1-Flag**), IAD-mutated vIRF1 (**vIRF1-MD2-Flag**), or its control (**pHAGE**). Cells were subjected to the anti-Myc immunoprecipitation and analyzed by Western blot using anti-Flag antibody. The relative intensities of the bands were quantified and normalized to Myc-tagged IRF3. The values are labeled under the protein bands. The relative values of proteins in the vIRF1-WT-Flag group were set as “1” for comparison. **(B).** HEK293T cells with IRF3 overexpression (**IRF3-Myc**) were transfected with the wild type vIRF1 (**vIRF1-WT-Flag**), the mutant vIRF1 (**vIRF1-K406R-Flag**, **vIRF1-K442R-Flag**, **vIRF1-MD2-Flag**), or its control (**pHAGE**). Cells were subjected to the anti-Myc immunoprecipitation and analyzed by Western blot using anti-Flag antibody. The relative intensities of the bands were quantified as in (**A**). **(C).** HEK293T cells with IRF3 overexpression (**IRF3-Myc**) were co-transfected with the wild type vIRF1 (**vIRF1-WT-Flag**), IAD-mutated vIRF1 (**vIRF1-MD2-Flag**) or its control (**pHAGE**), along with the p300 plasmid (**p300-HA**) or its control (**pcDNA3.1**). Cells were subjected to the anti-HA immunoprecipitation and analyzed by Western blot using anti-Myc antibody. The relative intensities of the bands were quantified and normalized to HA-tagged p300. The relative values of proteins in the p300-HA plus pHAGE group were set as “1” for comparison and labeled under the protein bands. **(D).** HEK293T cells with IRF3 overexpression (**IRF3-Myc**) were co-transfected with the wild type vIRF1 (**vIRF1-WT-Flag**), the mutant vIRF1 (**vIRF1-K406R-Flag**, **vIRF1-K442R-Flag**, **vIRF1-MD2-Flag**) or its control (**pHAGE**), along with the p300 plasmid (**p300-HA**) or its control (**pcDNA3.1**). Cells were subjected to the anti-HA immunoprecipitation and analyzed by Western blot using anti-Myc antibody. The relative intensities of the bands were quantified as in (**C**). **(E).** HEK293T cells with IRF3 overexpression (**IRF3-Myc**) were co-transfected with the wild type vIRF1 (**vIRF1-WT-Flag**), IAD-mutated vIRF1 (**vIRF1-MD2-Flag**) or its control (**pHAGE**), along with the CBP plasmid (**CBP-HA**) or its control (**pcDNA3.1**). Cells were subjected to the anti-HA immunoprecipitation and analyzed by Western blot using anti-Myc antibody. The relative intensities of the bands were quantified and normalized to HA-tagged CBP. The relative values of proteins in the CBP-HA plus pHAGE group were set as “1” for comparison and labeled under the protein bands. **(F).** HEK293T cells with IRF3 overexpression (**IRF3-Myc**) were co-transfected with the wild type vIRF1 (**vIRF1-WT-Flag**), the mutant vIRF1 (**vIRF1-K406R-Flag**, **vIRF1-K442R-Flag**, **vIRF1-MD2-Flag**) or its control (**pHAGE**), along with the CBP plasmid (**CBP-HA**) or its control (**pcDNA3.1**). Cells were subjected to the anti-HA immunoprecipitation and analyzed by Western blot using anti-Myc antibody. The relative intensities of the bands were quantified as in (**E**). **(G).** HEK293T cells with IRF3 overexpression (**IRF3-Myc**) were co-transfected with the vIRF1 plasmid (**vIRF1-WT-Flag**) or its control (**pHAGE**), the p300 plasmid (**p300-HA**) or its control (**pcDNA3.1**), along with the SIRT6 plasmid (**SIRT6-Myc**) or its control (**pCDH**). Cells were subjected to the anti-HA immunoprecipitation and analyzed by Western blot using anti-Myc antibody. The relative intensities of the bands were quantified and normalized to HA-tagged p300. The relative values of proteins in the p300-HA + pHAGE + pCDH group were set as “1” for comparison and labeled under the protein bands. **(H).** HEK293T cells with IRF3 overexpression (**IRF3-Myc**) were co-transfected with the vIRF1 plasmid (**vIRF1-WT-Flag**) or its control (**pHAGE**), the CBP plasmid (**CBP-HA**) or its control (**pcDNA3.1**), along with the SIRT6 plasmid (**SIRT6-Myc**) or its control (**pCDH**). Cells were subjected to the anti-HA immunoprecipitation and analyzed by Western blot using anti-Myc antibody. The relative intensities of the bands were quantified and normalized to HA-tagged CBP. The relative values of proteins in the CBP-HA + pHAGE + pCDH group were set as “1” for comparison and labeled under the protein bands.

A previous study has shown that vIRF1 interferes with IFN response and IRF3-mediated transactivation by competing for CBP/p300 co-activator recruitment [16]. Therefore, we determined whether SIRT6-mediated vIRF1 propionylation is essential for IRF3-CBP/p300 complex formation in the antiviral activities of IFN. Mutation of either Lys406 or Lys442 abolished the interaction between vIRF1 and IRF3 (Fig 5A and 5B). To further examine whether vIRF1 propionylation at Lys406 and Lys442 is linked to vIRF1 inhibition of the formation of IRF3-CBP/p300 complexes, Myc-tagged IRF3 and HA-tagged p300 or CBP were transiently co-transfected into HEK293T cells with the wild type or mutant vIRF1. As expected, wild type vIRF1 interacted with CBP/p300 co-activators to block its association with IRF3; however, mutation of any lysine in IAD (Fig 5C and 5E), particularly at Lys406 or Lys442, abolished the vIRF1 inhibitory effect (Fig 5D and 5F). Next, we examined the role of SIRT6-mediated vIRF1 propionylation in this process. Overexpression of SIRT6 significantly increased the amount of IRF3 immunoprecipitated p300 or CBP (Fig 5G and 5H).

**Fig 6:**
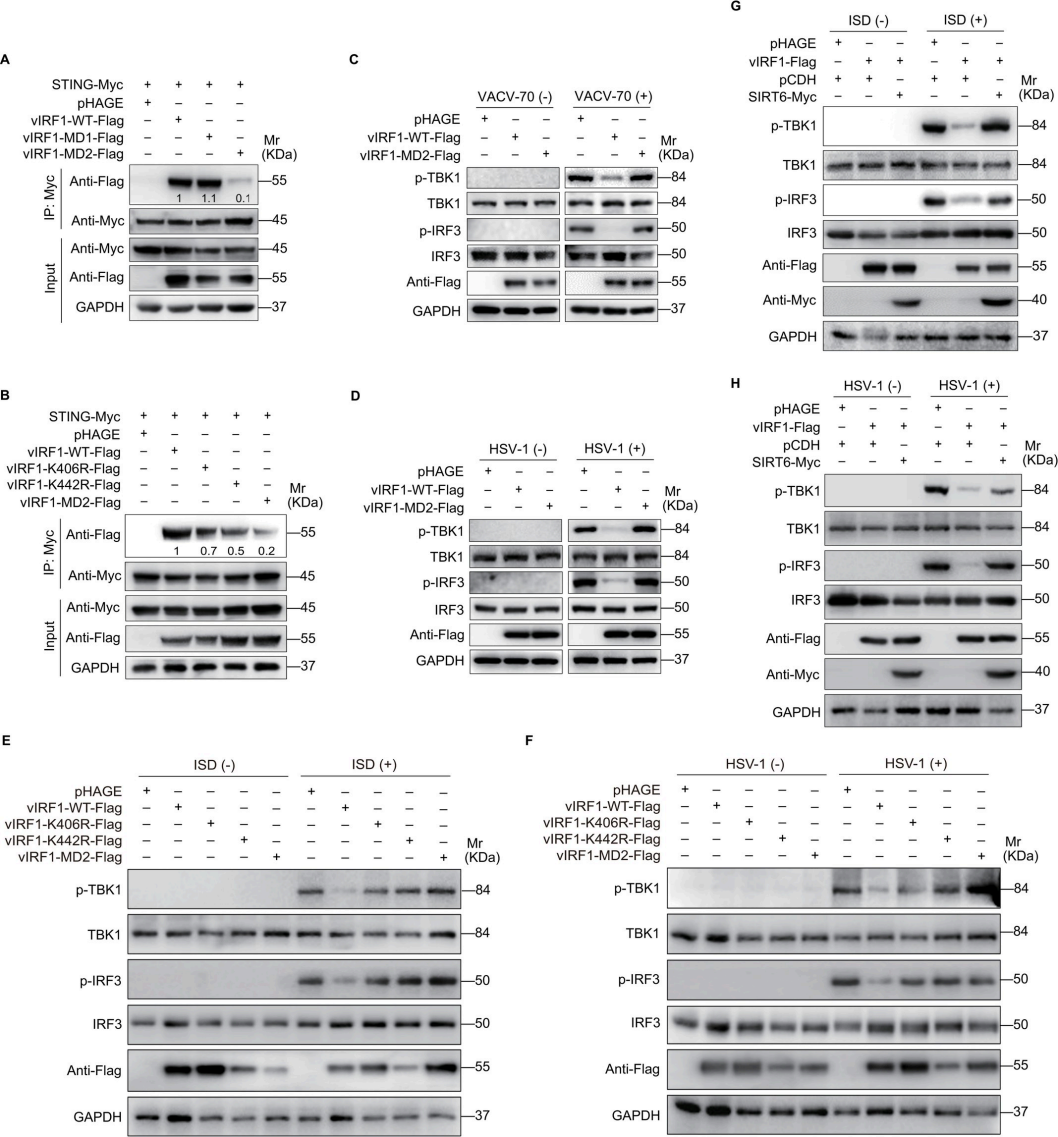
vIRF1 propionylation blocks the STING DNA sensing pathway. **(A).** HEK293T cells with STING overexpression (**STING-Myc**) were transfected with the wild type vIRF1 (**vIRF1-WT-Flag**), DBD-mutated vIRF1 (**vIRF1-MD1-Flag**), IAD-mutated vIRF1 (**vIRF1-MD2-Flag**), or its control (**pHAGE**). Cells were subjected to the anti-Myc immunoprecipitation and analyzed by Western blot using anti-Flag antibody. The relative intensities of the bands were quantified and normalized to Myc-tagged STING. The values are labeled under the protein bands. The relative values of proteins in the vIRF1-WT-Flag group were set as “1” for comparison. **(B).** HEK293T cells with STING overexpression (**STING-Myc**) were transfected with the wild type vIRF1 (**vIRF1-WT-Flag**), the mutant vIRF1 (**vIRF1-K406R-Flag**, **vIRF1-K442R-Flag**, **vIRF1-MD2-Flag**), or its control (**pHAGE**). Cells were subjected to the anti-Myc immunoprecipitation and analyzed by Western blot using anti-Flag antibody. The relative intensities of the bands were quantified as in (**A**). **(C-D).** EA.hy926 cells with the wild type vIRF1 (**vIRF1-WT-Flag**), IAD-mutated vIRF1 (**vIRF1-MD2-Flag**), or its control (**pHAGE**) overexpression were transfected with VACV-70 (**C**) or infected with HSV-1 (**D**). The phosphorylated TBK1 (**p-TBK1**), TBK1 (**TBK1**), phosphorylated IRF3 (**p-IRF3**) and IRF3 (**IRF3**) were examined by Western blot. **(E-F).** EA.hy926 cells with the wild type vIRF1 (**vIRF1-WT-Flag**), the mutant vIRF1 (**vIRF1-K406R-Flag**, **vIRF1-K442R-Flag**, **vIRF1-MD2-Flag**), or its control (**pHAGE**) overexpression were transfected with ISD90 (**E**) or infected with HSV-1 (**F**). The phosphorylated TBK1 (**p-TBK1**), TBK1 (**TBK1**), phosphorylated IRF3 (**p-IRF3**) and IRF3 (**IRF3**) were examined by Western blot. **(G-H).** EA.hy926 cells transduced with lentiviral vIRF1 (**vIRF1-Flag**) or its control (**pHAGE**) were infected with lentiviral SIRT6 (**SIRT6-Myc**) or its control (**pCDH**), and further transfected with ISD90 (**G**) or infected with HSV-1 (**H**). The phosphorylated TBK1 (**p-TBK1**), TBK1 (**TBK1**), phosphorylated IRF3 (**p-IRF3**) and IRF3 (**IRF3**) were measured by Western blot.

In addition to blocking IRF-3 recruitment of CBP/p300 co-activator, vIRF1 can also inhibit IFN-β activation through alternative mechanisms, such as by interacting with STING to suppress the phosphorylation of IRF3 and TBK1 in response to KSHV reactivation [17]. Thus, we examined the effect of vIRF1 propionylation on vIRF1 and STING interaction. Compared with wild type vIRF1, mutation of Lys406 or Lys442 alone, or all lysines in IAD, abolished vIRF1 binding to STING (Fig 6A and 6B). To determine the role of vIRF1 propionylation in the inhibition of STING-dependent IFN-β response, Western blot was performed to evaluate the phosphorylation and activation of TBK1 and IRF3 in EA.hy926 cells overexpressing wild type and mutant vIRF1. While wild type vIRF1 inhibited the phosphorylation of TBK1 and IRF3, mutation of Lys406 or Lys442 alone, or all lysines in IAD reversed this effect (Fig 6C–6F). Moreover, overexpression of SIRT6 reversed vIRF1 inhibition of phosphorylation of IRF3 and TBK1 in response to exogenous DNA or HSV-1 infection (Fig 6G and 6H).

Together these results indicate that vIRF1 propionylation inhibits the antiviral response of IFN by blocking IRF3-CBP/p300 recruitment and the STING DNA sensing pathway.

### SIRT6 activator UBCS039 promotes vIRF1 depropionylation to assist antiviral innate immunity

**Fig 7:**
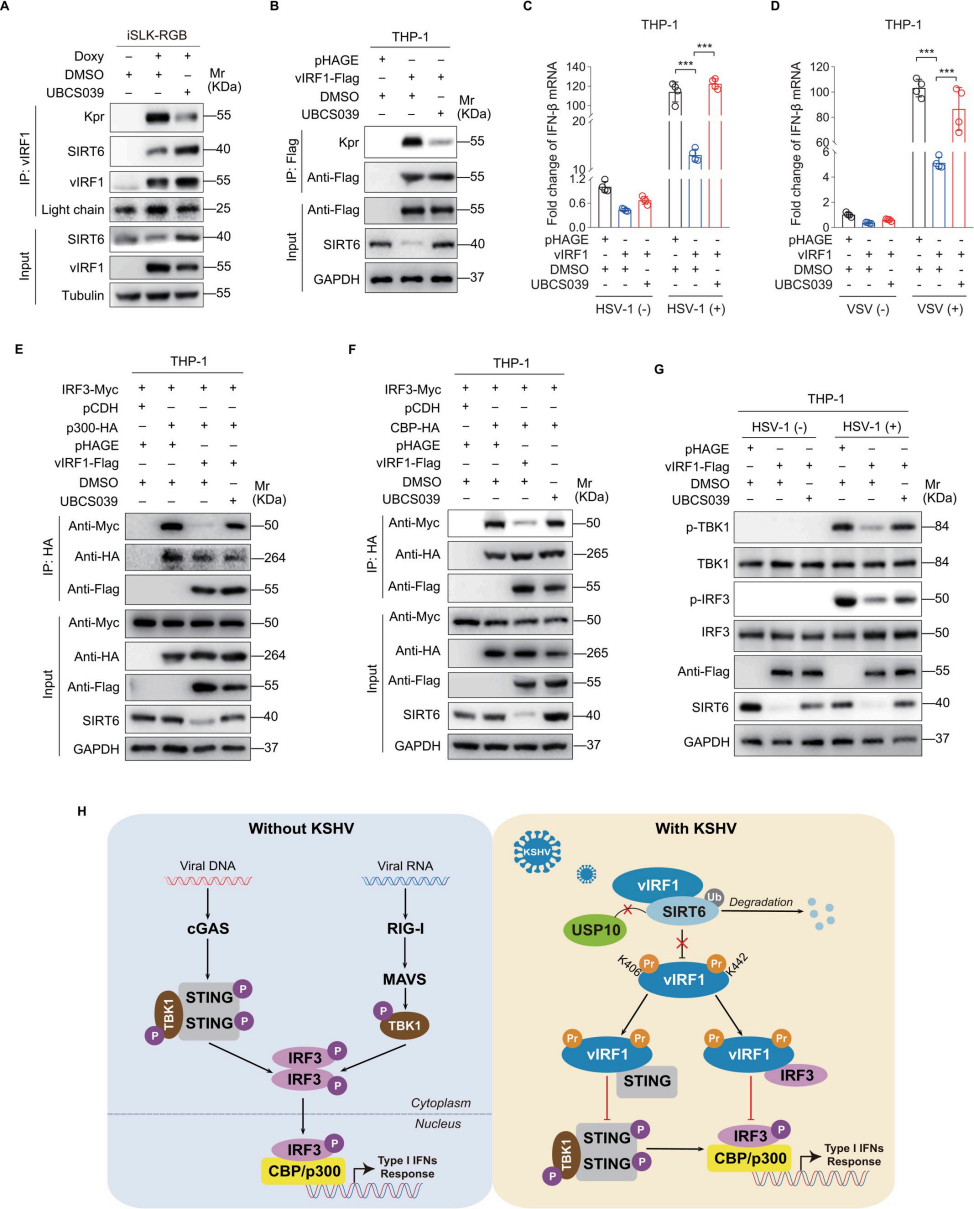
SIRT6 activator promotes vIRF1 depropionylation to assist antiviral innate immunity. **(A).** iSLK-RGB cells induced with or without Doxycycline (**Doxy**) for 48 h were treated with the SIRT6 activator UBCS039 (**UBCS039**; 80 μM) or its control (**DMSO**) for 24 h, and then subjected to the anti-vIRF1 immunoprecipitation. The immuno-isolated proteins were analyzed by Western blot using anti-propionyllysine (**Kpr**) antibody. **(B).** THP-1 cells transduced with lentiviral vIRF1 (**vIRF1-Flag**) or its control (**pHAGE**) were treated with the SIRT6 activator UBCS039 (**UBCS039**; 80 μM) or its control (**DMSO**) for 24 h, and then subjected to anti-Flag immunoprecipitation. The immuno-isolated proteins were analyzed by Western blot using anti-propionyllysine (**Kpr**) antibody. **(C-D).** THP-1 cells treated as in (**B**) were infected with HSV-1 (**C**) or VSV (**D**) for 16 h before IFN-β mRNA levels were detected by RT-qPCR. ***, *P* < 0.001 by Student’s *t* test. **(E).** THP-1 cells with IRF3 overexpression (**IRF3-Myc**) were transduced with lentiviral vIRF1 (**vIRF1-Flag**) or its control (**pHAGE**), p300 (**p300-HA**) or its control (**pCDH**), and further treated with the SIRT6 activator UBCS039 (**UBCS039**; 80 μM) or its control (**DMSO**) for 24 h. Cells were subjected to the anti-HA immunoprecipitation and analyzed by Western blot using anti-Myc antibody. **(F).** THP-1 cells with IRF3 overexpression (**IRF3-Myc**) were transduced with lentiviral vIRF1 (**vIRF1-Flag**) or its control (**pHAGE**), CBP (**CBP-HA**) or its control (**pCDH**), and treated with the SIRT6 activator UBCS039 (**UBCS039**; 80 μM) or its control (**DMSO**) for 24 h. Cells were subjected to the anti-HA immunoprecipitation and analyzed by Western blot using anti-Myc antibody. **(G).** THP-1 cells transduced with lentiviral vIRF1 (**vIRF1-Flag**) or its control (**pHAGE**) were treated with the SIRT6 activator UBCS039 (**UBCS039**; 80 μM) or its control (**DMSO**) for 24 h, and then infected with HSV-1. Cells were analyzed for the phosphorylated TBK1 (**p-TBK1**), TBK1 (**TBK1**), phosphorylated IRF3 (**p-IRF3**) and IRF3 (**IRF3**) levels by Western blot. (H). Schematic illustration for the mechanism of vIRF1 propionylation-involved immune evasion. KSHV vIRF1 prevents the interaction between SIRT6 and USP10 to promote the degradation of SIRT6, which serves as a depropionylase of vIRF1. Propionylated vIRF1 occurring on K406 and K442 blocks IRF3-CBP/p300 recruitment and the STING DNA sensing pathway, resulting in inhibition of type I IFN response.

We further explored SIRT6-mediated vIRF1 propionylation as potential target for inhibiting KSHV evasion of host immunity. UBCS039 is a selective activator of SIRT6. Treatment with UBCS039 in both iSLK-RGB and THP-1 cells not only enhanced SIRT6 expression but also depropionylated vIRF1 (Fig 7A and 7B). Furthermore, UBCS039 effectively increased the level of IFN-β transcript in response to virus infection (Fig 7C and 7D). UBCS039 activation of SIRT6 increased IRF3 recruitment of CBP/p300 co-activator that was blocked by propionylated vIRF1 (Fig 7E and 7F). Meanwhile, UBCS039 restored the levels of phosphorylated TBK1 and IRF3 suppressed by propionylated vIRF1 (Fig 7G). These results collectively indicate that SIRT6 activator UBCS039 promotes vIRF1 depropionylation leading to the recovery of host antiviral innate immunity.

## Discussion

Similar to phosphorylation, methylation and ubiquitination play critical roles in various biological processes, including immune defense against viruses. New advancements in mass spectrometry and biochemistry technologies have led to the discovery of many types of lysine acylation. Among them, lysine propionylation, known as covalent binding of a propionyl group (CH_3_-CH_2_-CO-) to lysine residues in proteins, was initially reported in histones, and later characterized as a transcriptionally active PTM in an *in vitro* system [23,24]. In 2009, Cheng et al. identified the first three non-histone protein substrates of lysine propionylation in eukaryotic cells, including p53, p300, and CBP [25]. However, whether propionylation exists in viral proteins is yet to be explored. Our study is the first to show that propionylation occurs in a viral protein, and this modification functionally regulates viral immune evasion.

Like other lysine acylations, propionylation is post-translational, reversible, and enzymatically regulated by the same set of acetyltransferases and deacylases. In eukaryotic cells, p300 and CBP, two previously known histone acetyltransferases (HATs), can catalyze propionyl transfer both *in vitro* and *in vivo* [23,25], and propionylation of CoA synthetase (propionyl-CoA synthetase) of *Salmonella* is catalyzed by the acetyltransferase Pat [26]. Interestingly, lysine deacylase SIRT1 has depropionylase activity in eukaryotic cells [25], and sirtuin-like deacetylase CobB regulates the propionylation of CoA synthetase in *Salmonella* [26], suggesting that sirtuins might function to remove propionyl groups. Therefore, we focused on the sirtuin family to screen the depropionylases for vIRF1. Noticeably, SIRT6 was shown to interact with vIRF1. A previous study showed the association between SIRT6 and KSHV DNA, which inhibited viral reactivation [27]. Here, we found that SIRT6 decreased the propionylation level of vIRF1, suggesting the potential enzymatic activity of SIRT6 in lysine propionylation *in vivo*. Therefore, vIRF1 may promote the degradation of depropionylase SIRT6 to prevent its inhibition of KSHV reactivation and immune escape.

As a highly conserved NAD^+^-dependent protein deacetylase, SIRT6 facilitates the removal of acyl groups from the ε-amino group of lysines [28]. Both activators and inhibitors have been developed for SIRT6 given its multiple roles of in human diseases. UBCS039, a newly synthesized pyrrolo[1,2-a] quinoxaline derivative, is the first synthetic activator and specific for SIRT6 deacetylase activity [29], which has been reported to trigger SIRT6-dependent autophagy in different types of human cancer cell lines [30], and to activate SIRT6 to shift the macrophages from M1 to M2 [31]. Interestingly, UBCS039 has been used to uncover the function of SIRT6 in the a mouse model of thioacetamide (TAA)-induced acute liver failure (ALF) [32]. Our study has shown that UBCS039 can effectively inhibit vIRF1 propionylation and impair vIRF1 suppression of IFN-β response.

Viral infection triggers multifaceted antiviral responses in the host, with the IFN system as the most potent. Viruses have co-evolved strategies to undermine host innate responses. KSHV evades innate immunity through encoding the viral homologues of IFN regulatory factors [14,15]. KSHV vIRFs have low amino acid homology with cellular IRFs and distinct spatial conformation. Similar to cellular IRFs, vIRF1 contains an N-terminal DBD and a C-terminal IAD [33]. However, since its DBD lacks three of five conserved tryptophan residues that are essential for DNA binding, vIRF1 loses its DNA-binding ability [14]. Instead, vIRF1 counters host innate immunity via direct interaction with cellular proteins [16,17,33–35]. Previous reports have shown that the N-terminal of vIRF1 is mainly responsible for its association with the constitutively active form of IRF3 [16], while multiple domains of vIRF1 and STING interact with each other [17]. In our study, mutations of all lysine residues in the IAD or a mutation at a critical propionylation site can dramatically block vIRF1-mediated recruitment of CBP/p300 coactivator by IRF3, as well as the binding of vIRF1 to STING, thereby relieving vIRF1 inhibition of the interferon signaling pathway. Two reasons may explain the difference between our results and those of previous studies. First, vIRF1 binding sites to IRF3 were identified based on the constitutively active form of IRF3 [IRF3(5D)], rather than the wide type form, by replacing the serine and threonine residues in the C-terminal domain of IRF3 with the phosphomimetic aspartic acid [36]. Thus, it cannot be ruled out that there is association between vIRF1 C-terminal domain and wild type IRF3. Second, the biological activities of proteins may be due to their three-dimensional (3D) structures and folding; therefore, the modification of amino acid residues may endow proteins with different structures and functions [37,38]. Similarly, the mutational perturbation of propionylated lysine residues in vIRF1 IAD domain may influence its structural properties, such as neighboring residues, secondary structure and surrounding hydrophobicity, which in turn affects subsequent formation of protein-protein complexes based on the residues at binding sites.

In the present study, although we characterized a novel acylation modification in a viral protein by combining mass spectrometry and lysine mutation analysis, the results obtained by the two methods were not exactly the same. For instance, Lys442 in vIRF1 has been fully proved to exhibit the functional role in vIRF1 propionylation and immune escape by lysine mutation analysis. However, we did not identify the propionylation Lys442 polypeptide in the mass spectrometry analysis. We speculated that the peptide was not protected from trypsin digestion, which resulted in its destruction. Moreover, besides propionylation, we also found an increased vIRF1 acetylation level. However, the lysine-to-arginine mutation in the DBD or IAD had no effect on vIRF1 acetylation. Using mass spectrometry, we only detected 2 potential acetylation sites (Fig 1D), in which the Lys2 located outside the functional domains showed a higher mass spectrometry score than that of Lys195. It may explain why the lysine mutation in vIRF1 functional domains alone did not influence its overall acetylation level. As the first newly discovered lysine acylation, propionylation is also structurally and functionally similar to acetylation, but the propionyl group is slightly larger than the acetyl group and may be functionally different. Several studies have demonstrated that the lysine acetylation and propionylation pathways share many substrates and regulatory enzymes. Indeed, we and others have discovered that several lysine-acetylation-regulating enzymes, such as SIRT6, SIRT1, p300 and CBP, also act on lysine propionylation [23,25]. How such modification achieves specificity in regulating different patterns of acylation modifiers remains unknown. The distinction of acetylation and propionylation at a particular lysine residue regulated by the same enzyme is also unclear. Finally, what is the functional difference between lysine acetylation and propionylation at the same substrate? As a relatively new identified modification, the understanding of propionylation is still limited by technologies and methods. Recent works have demonstrated that, acetylated and propionylated lysines, despite their similar structures, may lead to different protein-protein interactions and functions [20,39].

The innate immune response is the first line of defense against viral infections [40]. Viral DNAs and RNAs are sensed by cGAS and mitochondrial antiviral-signaling protein (MAVS) in the cytosol, respectively, and then signal via TBK1 to activate IRF3 [41,42]. Activated IRF3 enters the nucleus and binds to CBP/p300 co-activator, thereby triggering the expression of pro-inflammatory cytokines and type I IFNs. Our study has identified a novel mechanism of viral evasion of innate immunity through propionylating a viral protein. KSHV vIRF1 disrupts SIRT6 interaction with USP10 causing its degradation, hence stabilizing vIRF1 propionylation at K406 and K442, which is necessary for inhibiting IFN-β production by blocking IRF3-CBP/p300 recruitment and repressing the STING DNA sensing pathway (Fig 7H). Finally, we have found that an activator of SIRT6 UBCS039 is effective in inhibiting vIRF1 propionylation and enhancement of IFN-β signaling, which could potentially be explored for inhibiting KSHV infection and treatment of KSHV-related diseases.

## Materials and methods

### Cell culture and reagents

HEK293T cells and telomerase-immortalized human umbilical vein endothelial (TIVE) cells were maintained as previously described [43,44]. The human umbilical vein endothelial cell line EA.hy926 (#CRL-2922) and the human acute monocytic leukemia cell line THP-1 (#TCHu 57) were purchased from ATCC (Manassas, VA, USA) and National Collection of Authenticated Cell Cultures (Shanghai, China), respectively. HEK293T and EA.hy926 cells were cultured in Dulbecco’s modified Eagle’s medium (DMEM) containing 10% fetal bovine serum (FBS), while THP-1 cells were maintained in RPMI-1640 medium with 10% FBS. TIVE cells were grown in Medium 199 (10-060-CV, Corning) supplemented with 20% FBS, 60 μg/mL ECGF (E2759-5X15MG, Millipore Sigma), and 1% penicillin-streptomycin. iSLK-RGB cells and K9_mutant iSLK-RGB cells were kept in DMEM supplemented with 1.2 mg/mL hygromycin B (BBI, China), 250 μg/mL G418 (Biofroxx, China), 1 μg/mL puromycin (Beyotime, China) and 10% FBS [45]. All of cell lines were negative for mycoplasma by Myco-Blue Mycoplasma Detector (D103-01/02, Vazyme Biotech Co., Ltd, China) detection. Cycloheximide (CHX) was obtained from Sigma-Aldrich, and the proteasome inhibitor MG132 was from Selleck Chemicals (Shanghai, China). UBCS039 were purchased from MedChemExpress (HY-115453, China) and freshly dissolved in dimethylsulfoxide (DMSO) before treatment. VACV70 (#tlrl-vav70n), Poly I:C (#tlrl-pic-5) and interferon stimulatory DNA 90 (ISD90; #tlrl-isdn) were obtained from InvivoGen (Hong Kong, China).

### Plasmids and transfection

Based on the pHAGE-vIRF1-Flag expressing plasmid described in the previous studies [45], the vIRF1 domain-mutant plasmids (vIRF1-MD1-Flag, vIRF1-MD2-Flag), and the vIRF1 single lysine-mutant plasmids were constructed to replace the indicated lysine site(s) with arginine by Tsingke Biotechnology Co., Ltd. (Beijing, China), as well as the Lys406 and Lys442 co-mutant vIRF1 plasmid (vIRF1-K406R+K442R-Flag). The sirtuins expressing plasmids (SIRTs-1~7-Myc), USP10-Flag, IRF3-Myc and STING-Myc were generated based on the pCDH vector (Tsingke Biotechnology Co., Ltd., China). pcDNA3.1-p300-HA was kindly provided by Dr. Xiaoming Wang (Nanjing Medical University), and pcDNA3.1-CBP-HA was constructed based on a generous gift from Dr. Xiao Han (Nanjing Medical University). A 300 bp fragment of IFN-β promoter (-280 to +20) was amplified and subcloned into pGL3-basic vector containing the firefly luciferase reporter gene (Promega, USA). The mpCDH plasmid was used as the short hairpin RNA (shRNA) expressing lentiviral vector, and the shRNA sequences to USP10 were listed in S1 Table. All plasmids were prepared using Vazyme FastPure Plasmid Mini Kit and confirmed by DNA sequencing. HEK293T cells were transfected with Lipofectamine 2000 Reagent (Invitrogen, USA), and EA.hy926 cells was transfected with Effectence transfection reagent (Qiagen, China).

### Lentivirus, virus and infection

The packaging plasmid psPAX2 and the envelope plasmid pMD2.G were co-transfected with the lentivirus plasmids into HEK293T cells as previously described [46]. Lentivirus was collected to infect cells, and the infection efficiency was monitored by fluorescence microscopic examination. Herpes simplex virus type 1 (HSV-1) and vesicular stomatitis virus (VSV), both of which were kindly provided by Drs. Junjie Zhang and Ke Lan from Wuhan University, respectively, were collected from supernatants of infected Vero cells at the appearance of cytopathic effect (CPE). The viral titers in the supernatants were determined by standard plaque assay. HSV-1 and VSV were diluted in DMEM at multiplicity of infection (MOI) of 10 PFU/mL and collected at the indicated times post infection.

Production of KSHV was performed according to the previous study [47]. Briefly, the stable iSLK-BAC16 cells were induced with doxycycline (1 μg/mL) for 48 h, and then the maintaining medium was replaced for another 2 or 3 days. KSHV particles were pelleted of the cell supernatant through 20% sucrose cushion at 24 000 rpm for 3 h (4°C), and re-suspended in a desired volume.

### Co-immunoprecipitation (Co-IP) and mass spectrometry (MS)

Co-IP assay was performed as previously described [43]. Briefly, the IP lysis were centrifuged and incubated with 10 μL of anti-tagged immunomagnetic beads for overnight at 4°C. The beads were washed and eluted to collect the immunoprecipitated proteins for Western blot or MS analysis. For Western blot, the IPKine HRP Goat anti-mouse or anti-rabbit IgG LCS (Abbkine Scientific Co., Ltd, China) reacted with kappa light chains on IgG were utilized to avoid the detection of the heavy chains of IgG. The MS analysis was performed by Applied Protein Technology (Zhejiang, China).

### Western blot and antibodies

Western blot was performed as previously described [48]. Specific commercial primary antibodies used in Western blot was shown in S2 Table. Anti-vIRF1 rabbit polyclonal antibody was generated by immunization of rabbits (Abclonal, China). Generally, the recombinant vIRF1 protein (1–223 aa) was obtained though molecular cloning and prokaryotic expression system. Then the New Zealand white rabbits were immunized with the recombinant proteins and adjuvant for at least 4 times. After serum titer detection, the antibodies were collected by affinity or Protein A/G purification. The antibodies were validated by Western blot before use.

### RT-qPCR

Total RNA was isolated with TRIzol (Life Technologies, NY, USA), and then reverse transcribed into cDNA by HiScript III RT SuperMix (Vazyme Biotech Co., Ltd.). ChamQ SYBR qPCR Master Mix (Vazyme Biotech Co., Ltd.) on StepOnePlus Real-Time PCR System (Applied Biosystems) was used for RT-qPCR analysis according to the manufacturer’s instructions, and the results were further analyzed using the 2^-ΔΔCt^ method and standardized with GAPDH. The sequences of RT-qPCR primers were listed in S3 Table.

### Luciferase reporter assay

The IFN-β promoter reporter plasmid or the pGL3-basic construct was transfected into HEK293T cells, as well as the Renilla vector pRL-TK (Promega) for the transfection efficiency normalization. Relative luciferase activity was measured by the dual-luciferase reporter assay system obtained from Promega (Beijing) Biotech Co., Ltd.

### Enzyme-linked immunosorbent assay (ELISA)

The human IFN-β ELISA Kit (RK01630; ABclonal Technology Co.,Ltd., China) was used to detect the IFN-β level in cell culture supernatants. The absorbance was measured using a Microplate plate reader (BioTek, USA) set to 450 nm.

### Immunofluorescence assay (IFA)

vIRF1-Flag and SIRT6-Myc were transfected into HEK293T cells for 24 h to detect their co-localization, and the indicated cells were seeded on 12 mm diameter round glass coverslips in 24-well plates for overnight. Then, the cells were fixed with cold acetone for 15 min, permeabilized with 0.2% Triton X-100 for 15 min, blocked with 1% bovine serum albumin for 1 h, and incubated with the indicated primary antibodies and the corresponding secondary antibodies conjugated with Alexa Fluor fluorescent dyes (S2 Table). 4,6 diamidino-2-phenylindole (DAPI; Beyotime, China) were further incubated for 10 min, and images were observed with a confocal microscopy (Carl Zeiss, Freistaat Thü ringen, Germany).

### Statistical analysis

Numerical data are expressed as the mean±SD. Two group comparisons were analyzed using two-sided Student’s t-test. Differences with a *P* value of 0.05 or less were considered statistically significant. All the experiments were repeated at least for three times, unless otherwise stated.

## Supporting information

S1 Fig Mass spectrometry analysis of a vIRF1 peptide propionylated at Lys406. Propionylation of vIRF1 lysine residues on Lys406 (marked in red) was identified in HEK293T cells transduced with lentiviral vIRF1 by LC-MS/MS analysis. The b and y ions in the spectra of the peptide were marked in green and orange, respectively.
(TIF)

S2 Fig vIRF1 lysine mutation affects IFN-β transcript. HEK293T cells transduced with lentiviral wild type (**vIRF1-WT**) and the mutant forms (**vIRF1-K406R**, **vIRF1-K442R**, **vIRF1-K406R+K442R**) of vIRF1, or its control (**pHAGE**) were further infected with HSV-1 (**A**) or VSV (**B**) for 16 h before IFN-β mRNA levels measured by RT-qPCR. ***, *P* < 0.001 by Student’s *t* test.
(TIF)

S3 Fig KSHV infection promotes vIRF1 transcript. The transcript levels of vIRF1 in HEK293T and EA.hy926 cells infected with KSHV for 0, 8, 16, 24 h were examined by RT-qPCR. *, *P* < 0.05, **, *P* < 0.01, and ***, *P* < 0.001 by Student’s *t* test.
(TIF)

S4 Fig Neither KSHV infection nor vIRF1 overexpression affects SIRT6 transcripts. (**A**). The mRNA levels of SIRT6 in HEK293T and EA.hy926 cells infected with KSHV for 0, 8, 16 and 24 h were examined by RT-qPCR. *n*.*s*, not significant. (**B**). The mRNA levels of SIRT6 in HEK293T, EA.hy926 and TIVE cells transduced with lentiviral vIRF1 (**vIRF1**) or its control (**pHAGE**) for 48 h were examined by RT-qPCR. *n*.*s*, not significant.
(TIF)

S5 Fig USP10 overexpression enhances SIRT6 expression. The protein level of SIRT6 in HEK293T cells transfected with USP10 plasmid (**USP10-Flag**) or its control (**pCDH**) for 24 h was examined by Western blot.
(TIF)

S6 Fig Knockdown of USP10 inhibits SIRT6 expression. The proteins levels of USP10 and SIRT6 in HEK293T cells transduced with lentivirus-mediated short hairpin RNAs (shRNA) targeting USP10 (**shUSP10-1~3**), a mixture of USP10 shRNAs (**shUSP10 mix**) or its control (**mpCDH**) for 48 h were examined by Western blot.
(TIF)

S7 Fig Neither KSHV infection nor vIRF1 overexpression affects USP10 expression. (**A**). The protein level of USP10 in HEK293T cells transfected with vIRF1 plasmid (**vIRF1-Flag**) or its control (**pHAGE**) for 24 h was examined by Western blot. (**B**). The protein level of USP10 in HEK293T cells infected with KSHV for 0, 8, 16 and 24 h were examined by Western blot.
(TIF)

S1 Table The sequences of shRNAs. (DOCX)

S2 Table The source of antibodies. (DOCX)

S3 Table The sequences of specific primers for RT-qPCR. (DOCX)
