# Supplementary figures and images for: Immune evasion strategy involving propionylation by the KSHV interferon regulatory factor 1 (vIRF1)
Source: PLoS Pathog. 2023 Apr 6;19(4):e1011324. doi: 10.1371/journal.ppat.1011324 (PMC10112802; doi:10.1371/journal.ppat.1011324)

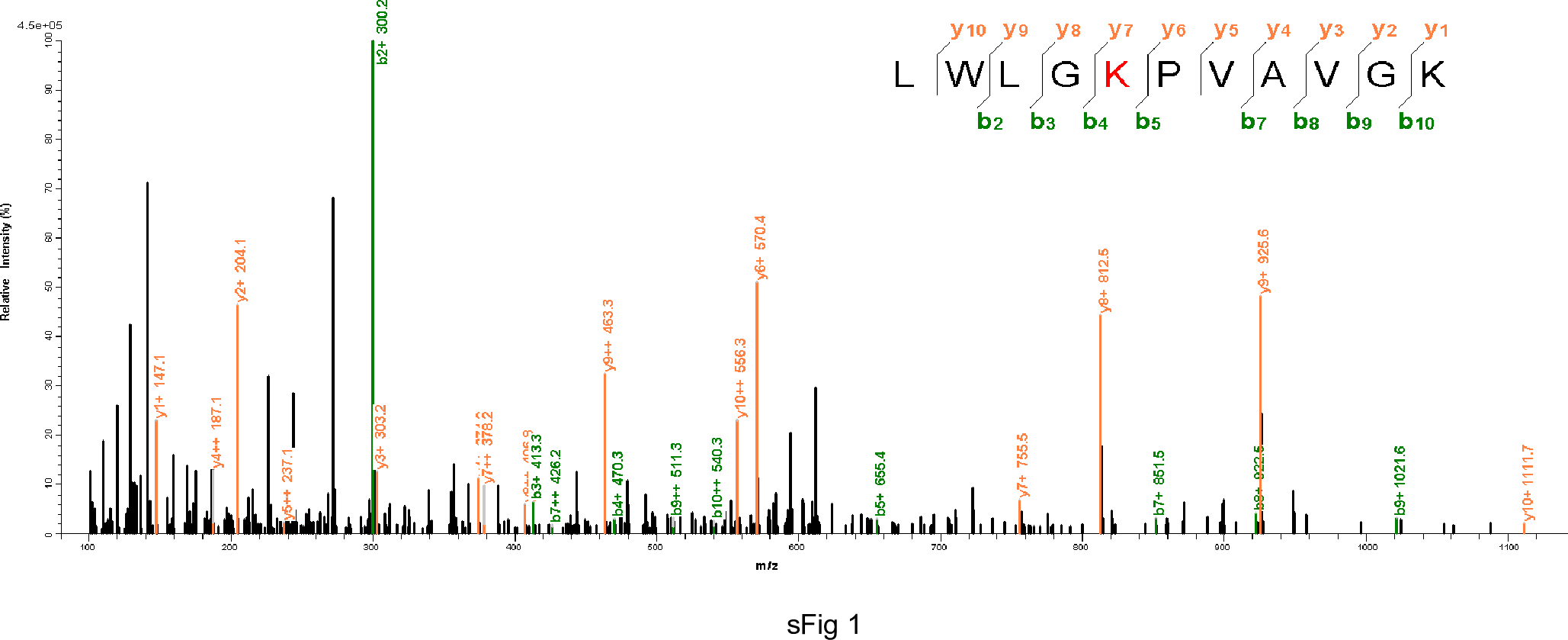

Supplement: S1 Fig — Propionylation of vIRF1 lysine residues on Lys406 (marked in red) was identified in HEK293T cells transduced with lentiviral vIRF1 by LC-MS/MS analysis. The b and y ions in the spectra of the peptide were marked in green and orange, respectively. (TIF) [file ppat.1011324.s001.tif]

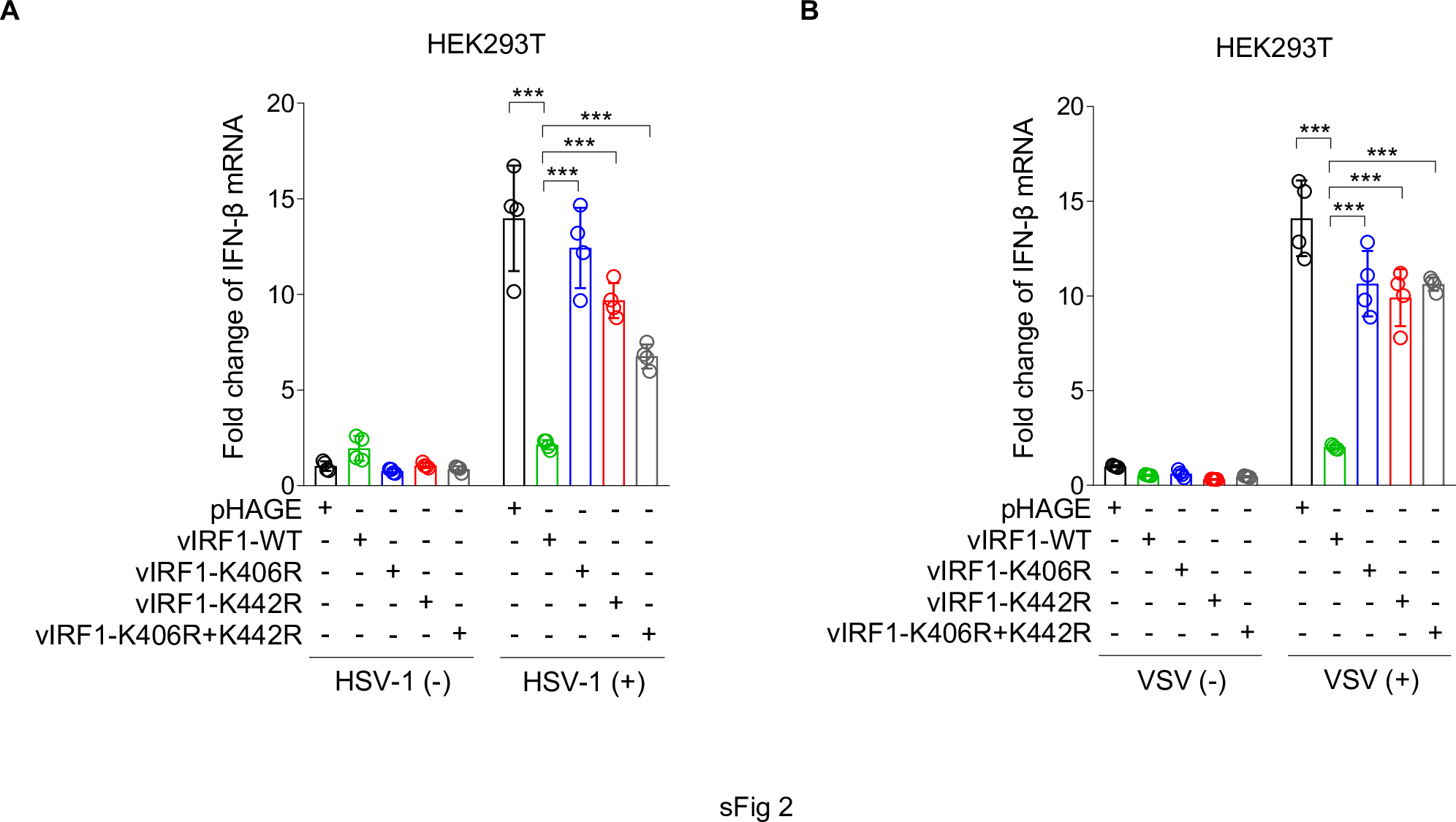

Supplement: S2 Fig — HEK293T cells transduced with lentiviral wild type (vIRF1-WT) and the mutant forms (vIRF1-K406R, vIRF1-K442R, vIRF1-K406R+K442R) of vIRF1, or its control (pHAGE) were further infected with HSV-1 (A) or VSV (B) for 16 h before IFN-β mRNA levels measured by RT-qPCR. ***, P < 0.001 by Student’s t test. (TIF) [file ppat.1011324.s002.tif]

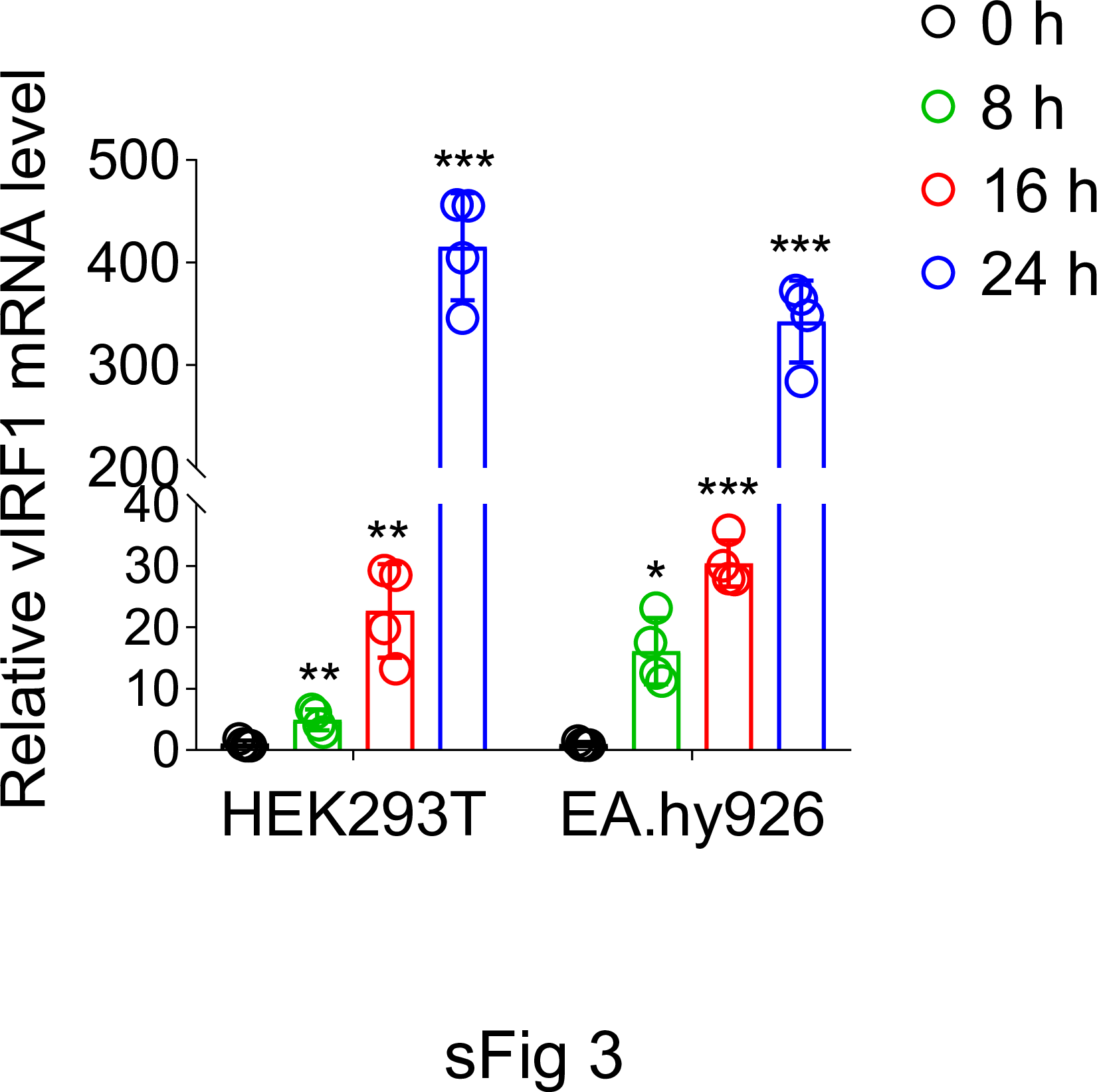

Supplement: S3 Fig — The transcript levels of vIRF1 in HEK293T and EA.hy926 cells infected with KSHV for 0, 8, 16, 24 h were examined by RT-qPCR. *, P < 0.05, **, P < 0.01, and ***, P < 0.001 by Student’s t test. (TIF) [file ppat.1011324.s003.tif]

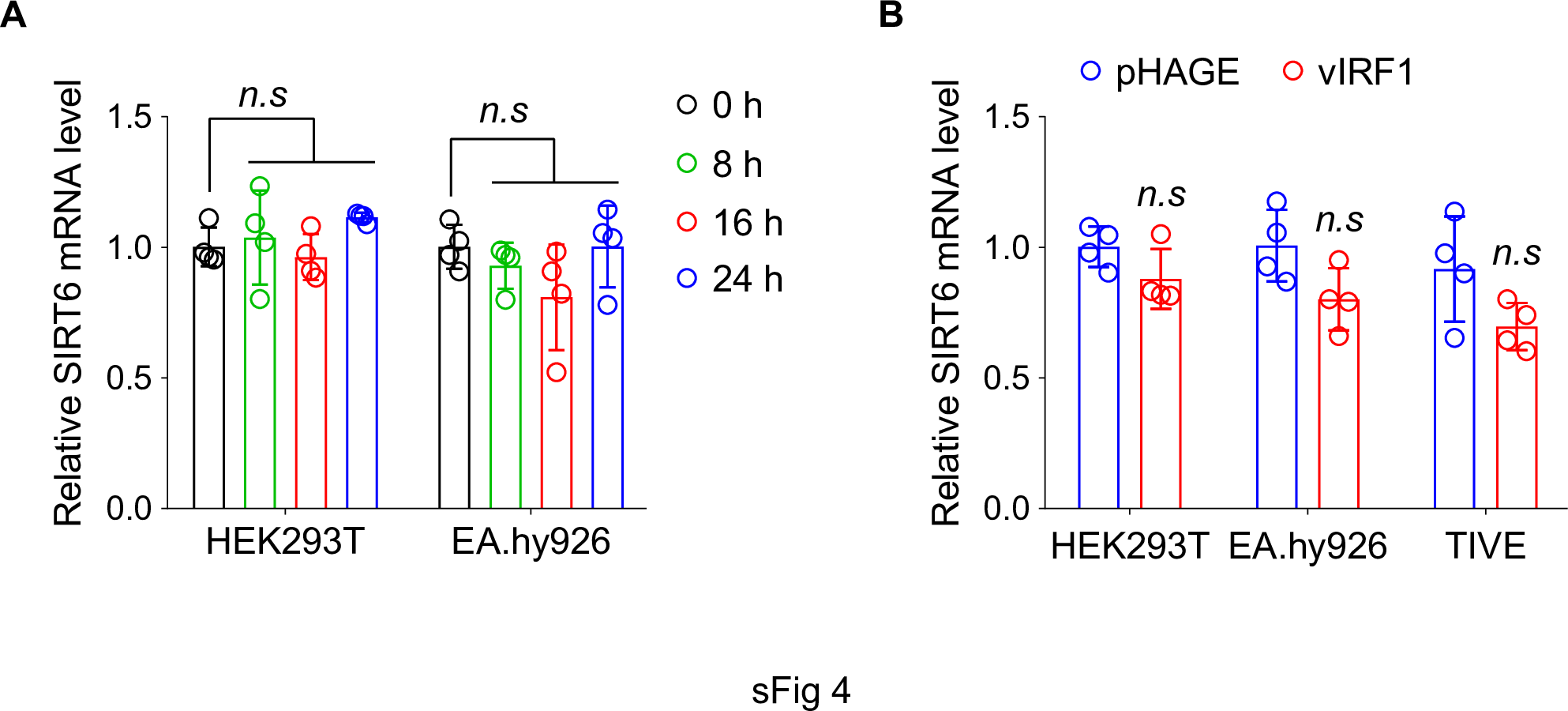

Supplement: S4 Fig — (A). The mRNA levels of SIRT6 in HEK293T and EA.hy926 cells infected with KSHV for 0, 8, 16 and 24 h were examined by RT-qPCR. n.s, not significant. (B). The mRNA levels of SIRT6 in HEK293T, EA.hy926 and TIVE cells transduced with lentiviral vIRF1 (vIRF1) or its control (pHAGE) for 48 h were examined by RT-qPCR. n.s, not significant. (TIF) [file ppat.1011324.s004.tif]

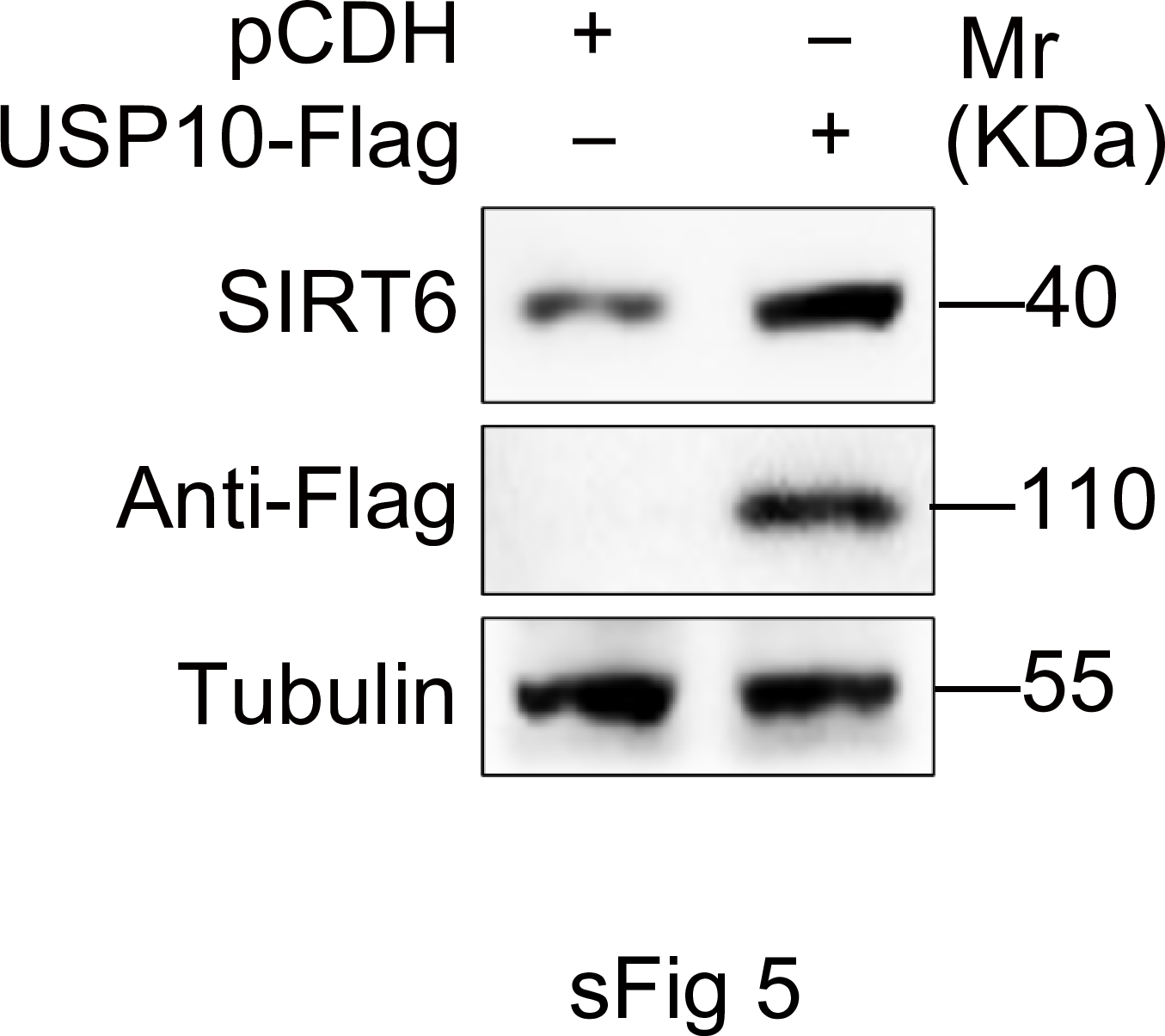

Supplement: S5 Fig — The protein level of SIRT6 in HEK293T cells transfected with USP10 plasmid (USP10-Flag) or its control (pCDH) for 24 h was examined by Western blot. (TIF) [file ppat.1011324.s005.tif]

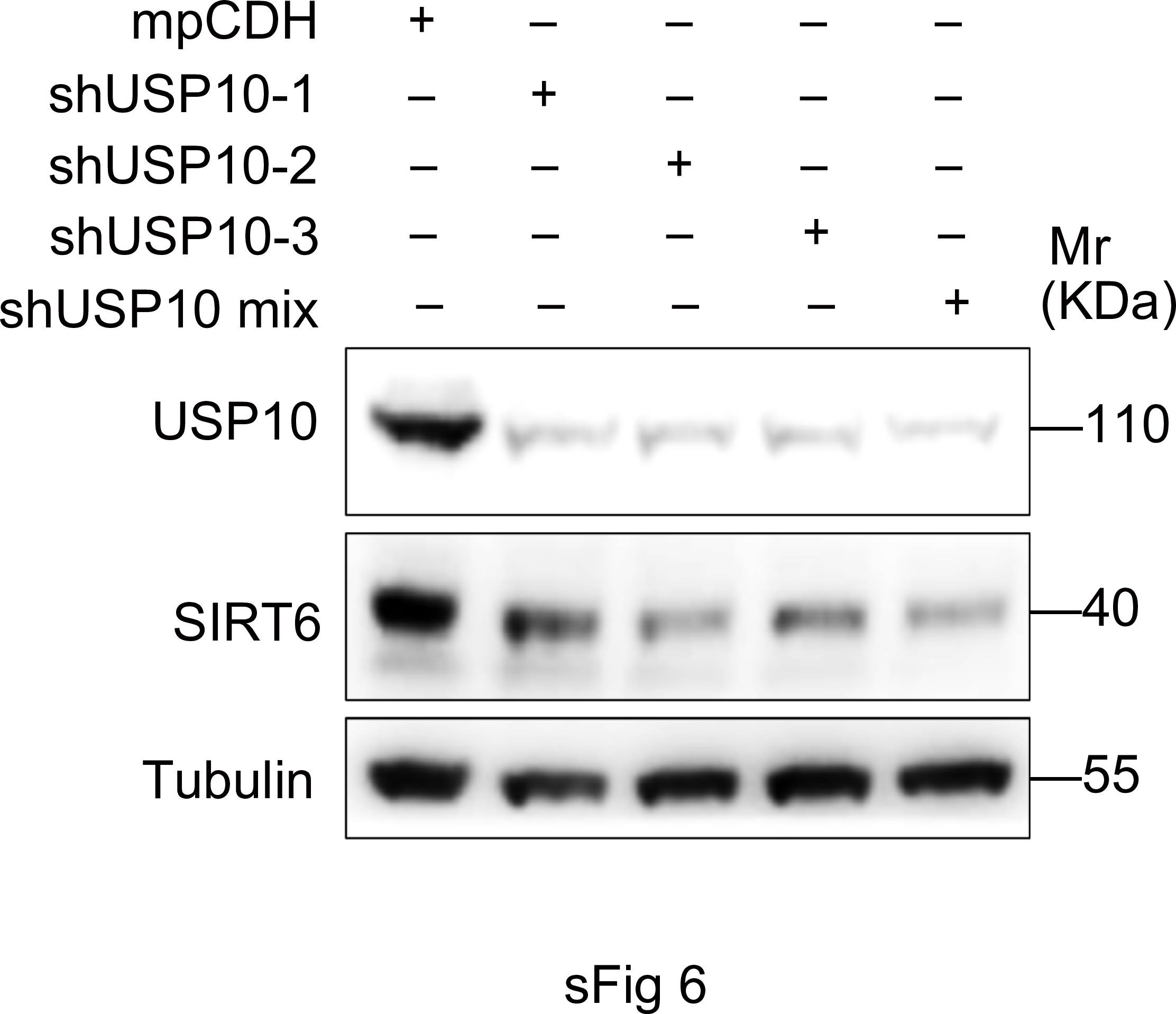

Supplement: S6 Fig — The proteins levels of USP10 and SIRT6 in HEK293T cells transduced with lentivirus-mediated short hairpin RNAs (shRNA) targeting USP10 (shUSP10-1~3), a mixture of USP10 shRNAs (shUSP10 mix) or its control (mpCDH) for 48 h were examined by Western blot. (TIF) [file ppat.1011324.s006.tif]

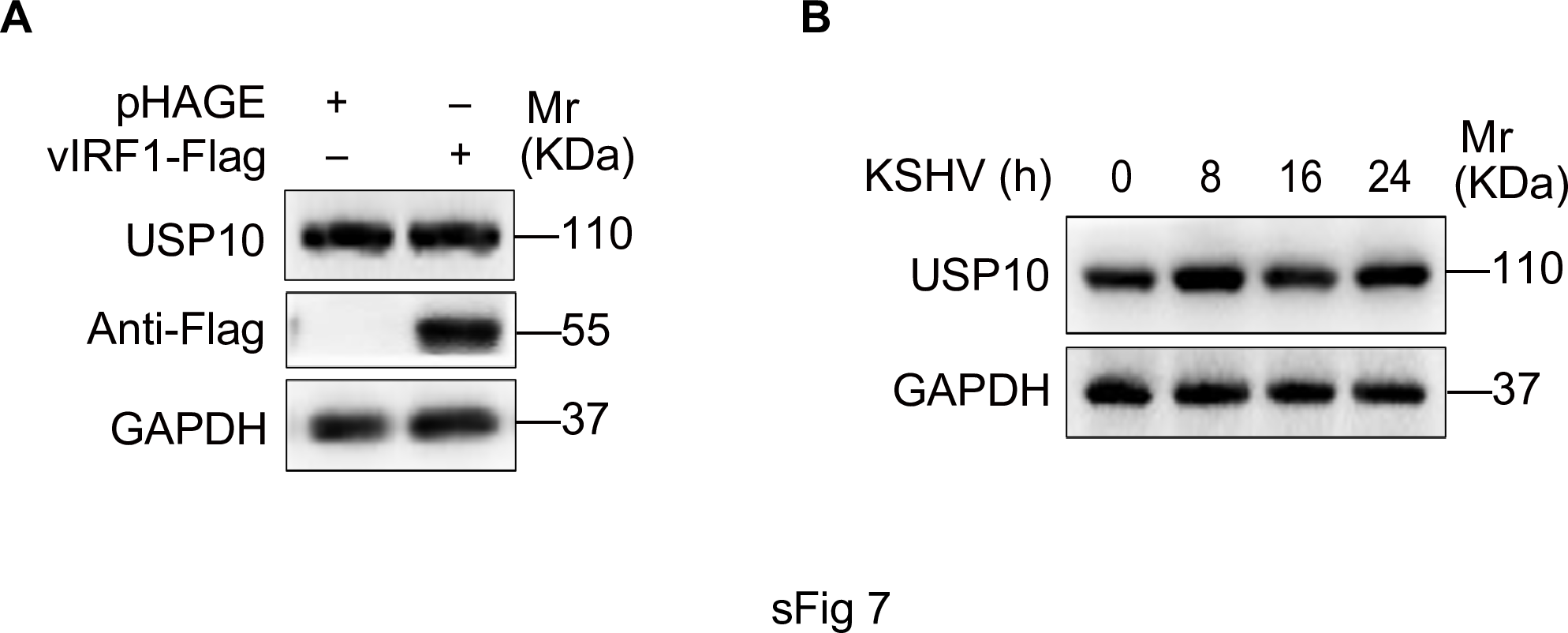

Supplement: S7 Fig — (A). The protein level of USP10 in HEK293T cells transfected with vIRF1 plasmid (vIRF1-Flag) or its control (pHAGE) for 24 h was examined by Western blot. (B). The protein level of USP10 in HEK293T cells infected with KSHV for 0, 8, 16 and 24 h were examined by Western blot. (TIF) [file ppat.1011324.s007.tif]
