# Supplementary material for: Immune evasion strategy involving propionylation by the KSHV interferon regulatory factor 1 (vIRF1)
Source: PLoS Pathog. 2023 Apr 6;19(4):e1011324. doi: 10.1371/journal.ppat.1011324 (PMC10112802; doi:10.1371/journal.ppat.1011324)
Supplement: S1 Table — (DOCX) [file ppat.1011324.s008.docx]

**S1 Table.** The sequences of shRNAs

| **shRNA** | **Sequence of shRNA (5’to 3’)** |
| --- | --- |
| shUSP10-1 | CACCTGAAACGATTCGTTTAT |
| shUSP10-2 | CGACAAGCTCTTGGAGATAAA |
| shUSP10-3 | CCTATGTGGAAACTAAGTATT |
