# Supplementary material for: Immune evasion strategy involving propionylation by the KSHV interferon regulatory factor 1 (vIRF1)
Source: PLoS Pathog. 2023 Apr 6;19(4):e1011324. doi: 10.1371/journal.ppat.1011324 (PMC10112802; doi:10.1371/journal.ppat.1011324)
Supplement: S2 Table — (DOCX) [file ppat.1011324.s009.docx]

**S2 Table.** The source of antibodies

| **Antibodies** | **Source** | **Identifier** |
| --- | --- | --- |
| anti-SIRT6 rabbit pAb | Proteintech | 13572-1-AP |
| anti-USP10 rabbit pAb | Proteintech | 19374-1-AP |
| anti-TBK1 rabbit pAb | Proteintech | 28397-1-AP |
| anti-IRF3 rabbit pAb | Proteintech | 11312-1-AP |
| anti-Flag rabbit pAb | Proteintech | 20543-1-AP |
| anti-p-TBK1 (Ser172) rabbit mAb | Cell Signaling Technology | 5483 |
| anti-p-IRF3 (Ser396) rabbit mAb | Cell Signaling Technology | 37829 |
| anti-α-tubulin mouse mAb | Santa Cruz | sc-23948 |
| anti-GAPDH mouse mAb | Santa Cruz | sc-47724 |
| anti-Flag mouse mAb | MEDICAL & BIOLOGICAL LABORATORIES CO., LTD. | M185-3L |
| anti-HA mouse mAb | MEDICAL & BIOLOGICAL LABORATORIES CO., LTD. | M180-3 |
| anti-Myc mouse mAb | MEDICAL & BIOLOGICAL LABORATORIES CO., LTD. | M192-3 |
| anti-propionyllysine rabbit pAb | Jingjie PTM BioLab | PTM-201 |
| anti-acetyllysine mouse mAb | Jingjie PTM BioLab | PTM-101 |
| anti-butyryllysine rabbit pAb | Jingjie PTM BioLab | PTM-301 |
| anti-crotonyllysine rabbit pAb | Jingjie PTM BioLab | PTM-501 |
| anti-2-hydroxyisobutyryllysine rabbit pAb | Jingjie PTM BioLab | PTM-801 |
| anti-β-hydroxybutyryllysine rabbit pAb | Jingjie PTM BioLab | PTM-1201 |
| anti-malonyllysine rabbit pAb | Jingjie PTM BioLab | PTM-901 |
| anti-succinyllysine rabbit pAb | Jingjie PTM BioLab | PTM-401 |
| Alexa Fluro 647-conjugated anti-rabbit IgG | Beyotime | A0468 |
| Alexa Fluro 555-conjugated anti-mouse IgG | Beyotime | A0460 |

pAb: polyclonal antibody; mAb: monoclonal antibody
