# Supplementary material for: Immune evasion strategy involving propionylation by the KSHV interferon regulatory factor 1 (vIRF1)
Source: PLoS Pathog. 2023 Apr 6;19(4):e1011324. doi: 10.1371/journal.ppat.1011324 (PMC10112802; doi:10.1371/journal.ppat.1011324)
Supplement: S3 Table — (DOCX) [file ppat.1011324.s010.docx]

**S3 Table.** The sequences of specific primers for RT-qPCR

| **Target** | **Primer Sequence** |
| --- | --- |
| IFN-β | F: 5’- ACTGCAACCTTTCGAAGCCT -3’ |
|  | R: 5’- AGCCTCCCATTCAATTGCCA -3’ |
| SIRT6 | F: 5’- ACTGCAACCTTTCGAAGCCT -3’ |
|  | R: 5’- AGCCTCCCATTCAATTGCCA -3’ |
| GAPDH | F: 5’- GAAGGTGAAGGTCGGAGTC -3’ |
|  | R: 5’- GAAGATGGTGATGGGATTTCC -3’ |

F, Forward; R, Reverse
